# Oncogenic zinc finger protein ZNF322A promotes stem cell-like properties in lung cancer through transcriptional suppression of c-Myc expression

**DOI:** 10.1038/s41418-018-0204-6

**Published:** 2018-09-26

**Authors:** Jayu Jen, Chun-Yen Liu, Yu-Ting Chen, Li-Ting Wu, Yang-Chih Shieh, Wu-Wei Lai, Yi-Ching Wang

**Affiliations:** 1 Department of Pharmacology, College of Medicine, National Cheng Kung University, Tainan, 70101 Taiwan; 2 Institute of Basic Medical Sciences, College of Medicine, National Cheng Kung University, Tainan, 70101 Taiwan; 3 Laura and Isaac Perlmutter Cancer Center, New York University School of Medicine, NYU Langone Health, New York, NY 10016 USA; 4 Division of Thoracic Surgery, Department of Surgery, National Cheng Kung University Hospital, College of Medicine, National Cheng Kung University, Tainan, 70101 Taiwan

**Keywords:** Cancer stem cells, Cancer metabolism, Oncogenes, Lung cancer, Prognostic markers

## Abstract

ZNF322A, a C2H2 zinc finger transcription factor, is an oncoprotein in lung cancer. However, the transcription mechanisms of ZNF322A in lung cancer stem cell-like reprogramming remain elusive. By integrating our chromatin immunoprecipitation-sequencing and RNA-sequencing datasets, we identified and validated the transcriptional targets of ZNF322A, which were significantly enriched in tumorigenic functions and developmental processes. Indeed, overexpression of ZNF322A promoted self-renewal ability and increased stemness-related gene expressions in vitro and in vivo. Importantly, ZNF322A bound directly to *c-Myc* promoter and recruited histone deacetylase 3 to transcriptionally suppress c-Myc expression, which in turn increased mitochondrial oxidative phosphorylation and promoted cell motility, thus maintaining stem cell-like properties of lung cancer. Clinically, ZNF322A^High^/c-Myc^Low^ expression profile was revealed as an independent indicator of poor prognosis in lung cancer patients. Our study provides the first evidence that ZNF322A-centered transcriptome promotes lung tumorigenesis and ZNF322A acts as a transcription suppressor of c-Myc to maintain lung cancer stem cell-like properties by shifting metabolism towards oxidative phosphorylation.

## Introduction

*ZNF322A*, which encodes a classical Cys2His2 zinc finger transcription factor, has been revealed as an oncogene in Asian and Caucasian lung cancer patients in our previous studies [1, 2]. Overexpression of ZNF322A transcriptionally dysregulates genes in control of cell growth and motility, therefore contributes to lung tumorigenesis and poor prognosis. For example, ZNF322A and c-Jun cooperatively bind to AP-1 elements on the *cyclin D1* and *alpha-adducin* promoters to transcriptionally upregulate their expression to enhance tumor growth and tumor metastasis [2]. However, the underlying transcriptional mechanisms and transcriptional networks of ZNF322A in lung tumorigenesis, especially reprogramming to cancer stem-like cells, remain elusive.

Growing lines of evidence have demonstrated the existence of cancer stem cells (CSCs), a small population of cells capable of self-renewal and differentiation, in solid tumors. CSCs have been shown to contribute to cancer relapse, chemotherapy resistance and distant organ metastasis [3–7]. Therefore, detailed mechanism of CSCs reprogramming emerges as an urgent issue and a promising strategy for anticancer therapy. Notably, ZNF322A mouse ortholog, Zfp322a, is revealed as a novel essential component of the transcription network for maintaining self-renewal and pluripotency of mouse embryonic stem (mES) cells [8]. Zfp322a promotes OKSM (Oct4, Klf4, Sox2, c-Myc)-induced mouse embryonic fibroblast reprogramming to mES cells by transcriptionally activating Oct4 and Nanog expression. These OKSM factors have also been reported to play critical roles in cancer stemness [9]. Therefore we hypothesized that ZNF322A in lung cancer may contribute to lung cancer stem cell-like (CSC-like) reprogramming through transcriptionally regulating stemness transcription factors, such as Oct4, Nanog, Sox2 and c-Myc.

In this study, we conducted genome-wide analyses including cell-based chromatin-immunoprecipitation sequencing (ChIP-seq) and RNA sequencing (RNA-seq) analyses and in vitro oligo binding assay to identify ZNF322A transcriptional targets and to characterize ZNF322A DNA binding elements. Gene ontology analysis of ZNF322A putative downstream genes elicited the contribution of ZNF322A in maintenance of CSC-like properties. Indeed, using lung cancer cell, xenograft and clinical models, we revealed a novel role of ZNF322A in lung CSC-like reprogramming maintenance and cell motility promotion through suppressing c-Myc expression at the transcriptional level causing a metabolic shift toward oxidative phosphorylation in lung cancer stem-like cells.

## Results

### Genome-wide mapping of ZNF322A binding sites in lung cancer cells

**Fig. 1:**
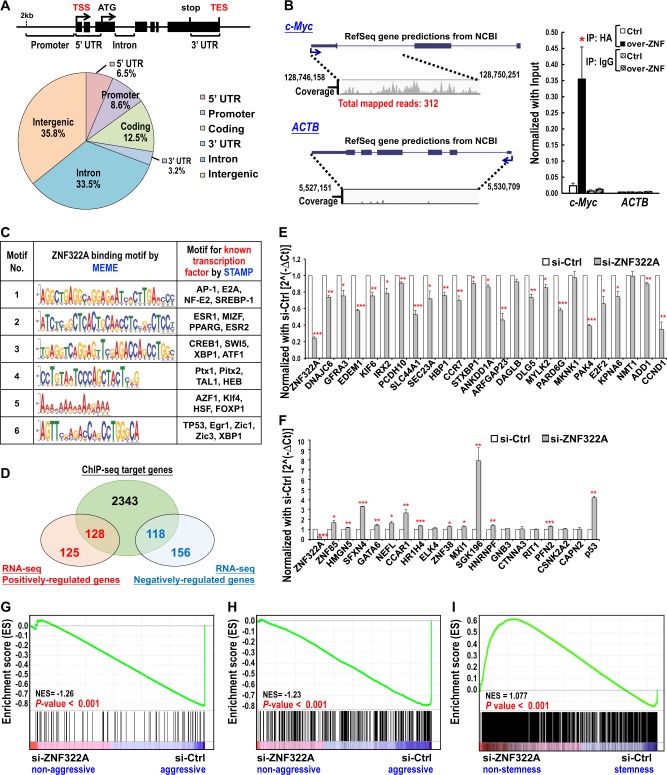
Establishment of ZNF322A transcriptome in lung cancer by integrating ChIP-seq and RNA-seq datasets. **a** Distribution of ZNF322A binding sites identified by ChIP-seq. Schematic diagram illustrates the definition of the location of a binding site in relation to a transcription unit (top panel). Pie diagram shows the location of ZNF322A binding sites relative to the nearest transcription unit (lower panel). **b** Snapshots of the ChIP-seq binding profiles of ZNF322A at *c-Myc* and *ACTB* genes from CLC Genomics Workbench are shown. The “coverage” represents the profile of ZNF322A binding (left). ChIP-qPCR analysis confirmed the HA-tagged ZNF322A (over-ZNF) occupancy at *c-Myc* gene. Cells transfected with empty vector are also shown (Ctrl). Normal IgG served as negative control. *ACTB* served as a negative control target gene (*right*). **c** ZNF322A binding elements were obtained by de novo motifs analysis using MEME software. The potential transcription factor binding elements related to ZNF322A targeting elements were analyzed by STAMP software. **d** ZNF322A transcriptional target genes were revealed by overlapping genes from RNA-seq and ChIP-seq. **e**, **f** Validation of RNA-seq datasets using qRT-PCR analysis for the expression of ZNF322A positively-regulated genes (**e**) and ZNF322A negatively-regulated genes (**f**) in cells transfected with si-control (si-Ctrl) or si-*ZNF322A* oligos. *GAPDH* gene served as internal control. Data are mean ± SEM. *P* values were determined using two-tailed Student’s *t*-test (*P *<* 0.05; **P *<* 0.01; ***P *<* 0.001). **g**–**i** GSEA analysis showed that the expression profile in si-ZNF322A lung cancer cells was significantly and negatively enriched with lung cancer expression profiles from human lung carcinoma tissue gene set [16] (**g**), the human lung cancer cell gene set [Wooster et al., unpublished data deposited in Oncomine database] (**h**), and the zfp322a silenced mouse embryonic stem cells [8] (**i**)

ZNF322A is an oncogenic transcriptional factor in lung cancer. However, the genomic binding pattern of ZNF322A in lung cancer is still unknown and uncovering it could reveal how ZNF322A contribute to lung tumorigenesis. To this end, we determined the genome-wide target sites of ZNF322A in H460 lung cancer cells expressing HA-ZNF322A using ChIP-seq approach. Based on two biological replicates, a total of 3666 ChIP regions corresponding to 2343 unique RefSeq genes were identified. Interestingly, we found that ZNF322A binding sites located in the 5’UTR, promoter, coding region and 3’UTR of the transcription unit, which accounted for 30.7% of total reads (Fig. 1a). In particular, ZNF322A was found bound at the promoter and gene body region of *c-Myc* for the first time (left, Fig. 1b). ChIP-qPCR confirmed the binding of ZNF322A at *c-Myc* gene locus in H460 cells overexpressing HA-ZNF322A (right, Fig. 1b). *ACTB* was included as a negative control in ChIP-qPCR validation. Additional 18 putative ZNF322A targeting sites were all cross-validated by ChIP-qPCR (Supplementary Figure 1A). Many of the validated genes bound by ZNF322A such as *HMGN5*, *GATA6*, *CCAR1* and *ELK4*, were found to be involved in tumorigenesis in other cancers [10–13], further supporting the oncogenic role of ZNF322A.

Next, we attempted to identify the DNA binding elements of ZNF322A. We adopted MEME software [14] for de novo motif analysis followed by STAMP software analysis [15] to identify the genomic DNA sequences and the potential ZNF322A-interacting transcription factors that may mediate the preferential recruitment of ZNF322A. As shown in Fig. 1c, we identified not only the potential DNA binding elements of ZNF322A but also binding motifs predicted to be the binding sites for transcription factors such as AP-1, CREB1, FOXP1, and TP53. Of note, ZNF322A top second MEME motif was found to play a crucial role in transcription regulation of *c-Myc* (data shown in *c-Myc* section). In addition, nine rounds of in vitro GST-ZNF322A pulldown cyclic amplification and selection of targets (CASTing) assay was conducted to confirm the DNA binding elements of ZNF322A. Interestingly, the top four elements identified in CASTing were found similar with the top fourth, fifth, second, third elements identified in ChIP-seq, respectively (Supplementary Figure 1B). Our ChIP-seq and in vitro CASTing assays confirmed that ZNF322A directly interacts with specific DNA elements and revealed putative canonical DNA binding sites of ZNF322A.

### Identification of ZNF322A transcriptional targets by integrating ChIP-seq and RNA-seq analyses

To further verify the transcriptional target genes directly bound by ZNF322A, we performed RNA-seq in ZNF322A knockdown cells followed by integration with ChIP-seq. Among ZNF322A differentially regulated genes from RNA-seq dataset, 128 positively-regulated genes and 118 negatively-regulated genes were identified in ChIP-seq (Fig. 1d). We validated 24 ZNF322A positively-regulated genes and 19 ZNF322A negatively-regulated genes by qRT-PCR. Among ZNF322A positively-regulated genes, the expression of 21 downstream targets were downregulated upon ZNF322A knockdown (Fig. 1e). On the other hand, 13 out of 19 ZNF322A negatively-regulated genes were upregulated upon ZNF322A knockdown (Fig. 1f). The validation rate for RNA-seq was 79.1%.

To characterize the importance of ZNF322A-driven transcriptome in cancer progression, we used MetaCore to analyze disease biomarker enrichment and the result showed the potential tumorigenic function of ZNF322A in various cancers, including breast, prostate, ovarian and colorectal cancers (Supplementary Figure 2A). Moreover, we conducted gene set enrichment analysis (GSEA) to determine whether ZNF322A-silenced transcriptome alteration in the context of lung cancer was inversely correlated with lung cancer datasets in lung carcinoma tissues [16] or lung cancer cell lines (Wooster et al., not published) deposited in Oncomine. Results showed that upregulated genes upon ZNF322A knockdown were enriched in non-aggressive gene group while downregulated genes upon ZNF322A knockdown were enriched in aggressive gene group in lung cancer analyzed by these two datasets (Fig. 1g, h).

### ZNF322A overexpression drives and maintains lung cancer stemness-like properties

Notably, ZNF322A mouse ortholog Zfp322a possesses essential roles in early development of mouse embryonic stem (mES) cells [8]. We thus hypothesized that ZNF322A could drive the conversion of somatic cancer cell into CSC-like state. Indeed, GSEA analysis showed a significant enrichment between the expression profiles in ZNF322A silenced A549 cells and in Zfp322a silenced mES cells (Fig. 1i). In addition, gene ontology analyses using DAVID (Database for Annotation, Visualization, and Integrated Discovery) bioinformatics software for ChIP-seq data revealed that ZNF322A downstream targets were significantly enriched in morphogenesis and development (Supplementary Figure 2B).

**Fig. 2:**
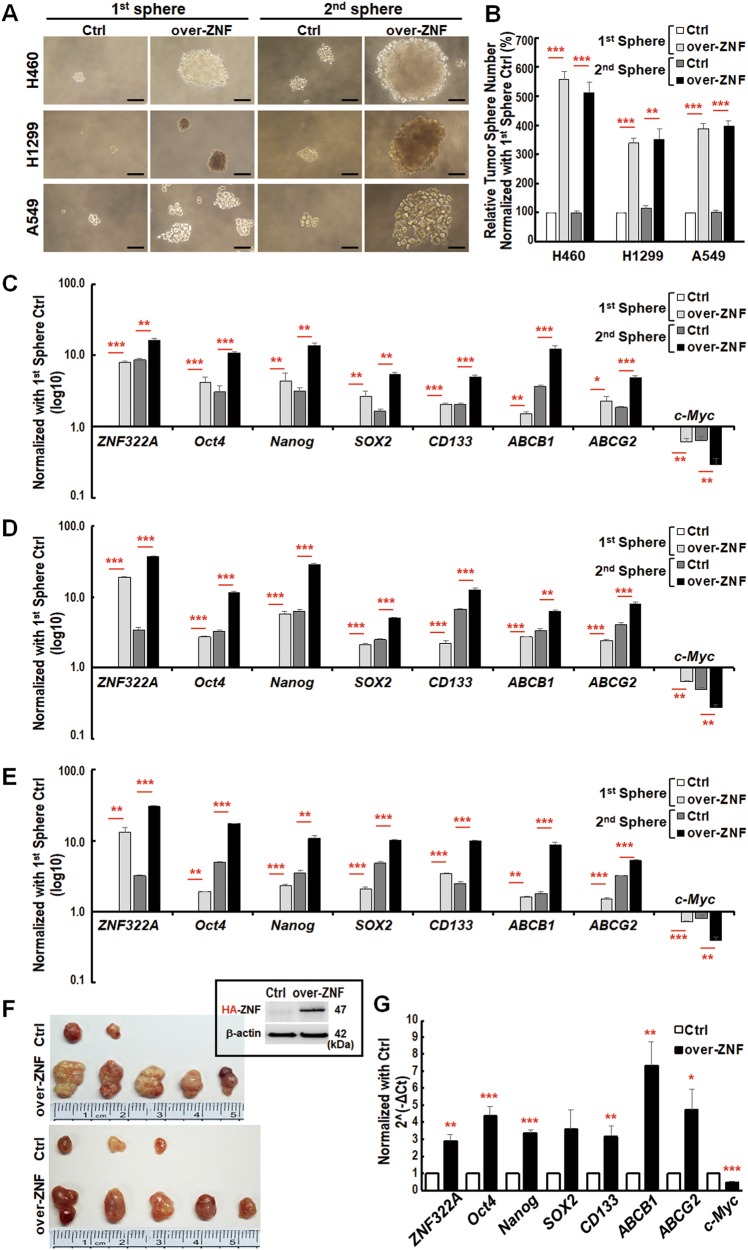
Ectopic expression of ZNF322A induces self-renewal and stemness-related gene expression in lung cancer cells in vitro and in vivo. **a**, **b** In vitro tumor sphere formation assay of H460, H1299 and A549 lung cancer cells overexpressing HA-tagged ZNF322A (over-ZNF) or empty vector (Ctrl) were photographed (**a**) and quantified (**b**). Scale bar, 200 nm. **c**–**e** qRT-PCR analysis of stemness-related genes and ZNF322A expression level in Ctrl or over-ZNF H460 (**c**), H1299 (**d**) or A549 (**e**) lung cancer cells. **f** Limited cell number (500 cells) of Ctrl or over-ZNF H460 sphere cells were mixed with matrigel and subcutaneously injected into BALB/c nude mice. Tumors were photographed after 28 days of implantation. ZNF322A overexpression in H460 cells was confirmed using immunoblotting (inset). **g** qRT-PCR analysis of stemness-related genes and ZNF322A expression level in H460 xenograft obtained from (**f**). *GAPDH* was used as internal control. The error bars represent SEM from three independent experiments (*P *<* 0.05; **P < 0.01; ***P *<* 0.001)

Since ZNF322A bound strongly to *c-Myc*, a transcription factor that reprograms mES, in our ChIP-seq dataset (Fig. 1b), we next validate if ZNF322A contributes to CSC-like formation and maintenance in lung cancer. To this end, we performed in vitro sphere formation assay, in vivo tumor initiation assay and examined stemness markers expression upon manipulation of ZNF322A expression in various lung cancer cells. Indeed, ZNF322A overexpression enhanced sphere formation abilities in H460, H1299 and A549 cells (Fig. 2a, b). The mRNA expression of *ZNF322A* and stemness markers, including *Oct4*, *Nanog*, *Sox2*, *CD133*, *ABCB1* and *ABCG2*, were found to be gradually increased upon ZNF322A overexpression in monolayer, first-sphere and second-sphere of H460, H1299 and A549 cells (Fig. 2c–e). On the other hand, ZNF322A knockdown suppressed the sphere formation abilities in H460, H1299, and A549 cells (Supplementary Figure 3A, B). Consistently, the mRNA expression of *Oct4*, *Nanog* and *CD133* were found downregulated upon ZNF322A knockdown (Supplementary Figure 3C, D).

Furthermore, in vivo tumor initiating assay was conducted to verify the role of ZNF322A in CSC-like formation and maintenance. After 28 days of implantation, 10 out of 10 immunodeficient mice injected with H460 first-sphere cells overexpressing ZNF322A (500 cells) bore large tumor burden, whereas 5 out of 10 formed small tumors in vector control sphere cells (Fig. 2f). Stemness-related genes and *ZNF322A* were also increased in ZNF322A overexpressed tumors compared to that in vector control (Fig. 2g). Our data indicated that enforced ZNF322A expression drove somatic cancer to CSC-like states such as self-renewal and differentiation in suspension cultures and at the limit number of xenotransplantation modeling.

### ZNF322A negatively regulates c-Myc expression at the transcription level

**Fig. 3:**
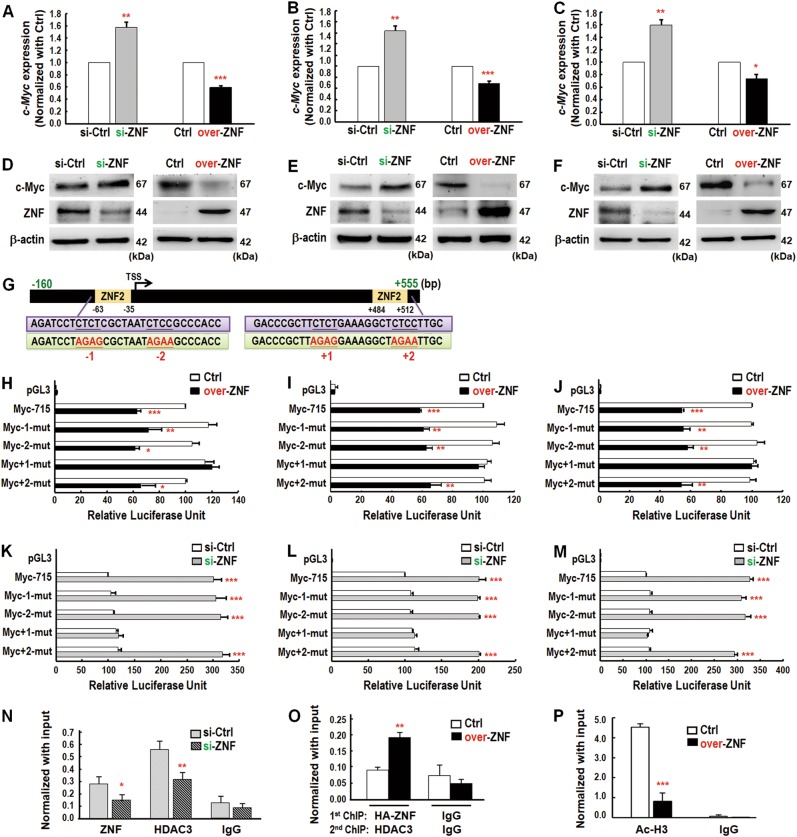
ZNF322A negatively regulates c-Myc expression at transcriptional level. **a**–**c** qRT-PCR analysis of *c-Myc* mRNA expression level in si-ZNF or over-ZNF H460 (**a**), H1299 (**b**) and A549 (**c**) lung cancer cells. **d**–**f** Immunoblotting analysis of c-Myc protein expression level in si-ZNF or over-ZNF H460 (**d**), H1299 (**e**), and A549 (**f**) lung cancer cells. Molecular weight endogenous ZNF322A is 44 kDa and HA-tagged ZNF322A is 47 kDa. **g** Minimal promoter region of *c-Myc* (-160/ + 555) contained two putative ZNF322A top second-MEME motifs (ZNF2, −63~−35 and +484~+512). Highly conserved sequences were mutated by site-directed mutagenesis and indicated as −1, −2, +1, and +2 (red text). **h**–**m** Dual luciferase activity assays were performed using firefly luciferase reporter vectors containing −160/+555 fragments of *c-Myc* promoters (Myc-715) or mutant ZNF322A binding motif of *c-Myc* promoters (Myc-1-mut, Myc-2-mut, Myc + 1-mut and Myc + 2-mut). Data represent promoter activity in Ctrl or over-ZNF H460, H1299 and A549 cells (**h**–**j**) or in si-Ctrl or si-ZNF cells (**k**–**m**). *TSS* transcription start site. **n**–**p** ZNF322A recruits HDAC3 and modulates histone acetylation (Ac-H3) on *c-Myc* promoter region. ChIP-qPCR and sequential ChIP-qPCR analyses were performed in si-Ctrl or si-ZNF (**n**) or Ctrl or over-ZNF cells (**o**, **p**). qPCR products of the target *c-Myc* promoter region relative to input are indicated on the Y-axis and antibodies for the proteins analyzed are on the X-axis. IgG serves as a negative control. The error bars represent SEM from three independent experiments (*P *<* 0.05; **P < 0.01; ***P *<* 0.001)

We noticed that *c-Myc* mRNA expression was decreased in ZNF322A-overexpressing first-sphere and second-sphere lung cancer cells (Fig. 2c–e) and sphere-derived xenograft tissues (Fig. 2g). In line with our results, increased *c-myc* expression was also evident in the expression microarray data of Zfp322a silenced mES cells [8]. To further confirm the negative regulation of ZNF322A on c-Myc expression, qRT-PCR and immunoblotting assays were conducted and the results confirmed that ZNF322A negatively regulated c-Myc mRNA and protein expression in ZNF322A-overexpressed or silenced H460 (Fig. 3a, d), H1299 (Fig. 3b, e) and A549 cells (Fig. 3c, f).

Next, we investigated the underlying mechanism by which ZNF322A transcriptionally downregulated *c-Myc*. We first analyzed the *c-Myc* promoter activities of various lengths of *c-Myc* promoter regions, including −2712/+555, −1715/+555 and −160/+555. The results showed that ZNF322A overexpression suppressed the activities of all three *c-Myc* promoters, whereas ZNF322A knockdown enhanced the activities of all three *c-Myc* promoters (Supplementary Figure 4). Further experiments were then conducted using the minimal promoter region of *c-Myc* (−160/+555). As revealed by the MEME motif analysis in Fig. 1c and shown by the map in Fig. 3g, ZNF322A top second-MEME motif (ZNF2) was found at −63/−35 and +484/+512 on the minimal *c-Myc* promoter. Luciferase promoter activity assay combined with site-directed mutagenesis at −1, −2, +1, and +2 DNA elements were performed by changing CTCT and CTCC (black text) to AGAG and AGAA (red text) sequences, respectively (Fig. 3g). Our results showed that ZNF322A overexpression suppressed *c-Myc* promoter activity but did not suppress *c-Myc* promoter with mutation at +1 DNA element (defined as *Myc* + *1-mut*) (Fig. 3h–j). Notably, knockdown of ZNF322A increased *c-Myc* promoter activity, while *Myc* + *1-mut* promoter activity was abolished upon ZNF322A knockdown in H460, H1299 and A549 cells (Fig. 3k–m).

In our previous study, we have shown that ZNF322A recruits histone deacetylase 3 (HDAC3) to suppress *p53* transcription [2]. This prompted us to investigate the interplay between ZNF322A and HDAC3 on *c-Myc* promoter by ChIP-qPCR assays. Interestingly, ZNF322A knockdown not only decreased ZNF322A binding but also significantly attenuated HDAC3 targeting to the *c-Myc* promoter (Fig. 3n). Consistently, re-ChIP results supported the co-occupancy of ZNF322A and HDAC3 at the *c-Myc* promoter (Fig. 3o). In addition, the active histone marker acetylated histone 3 (Ac-H3) was found to be decreased on the *c-Myc* promoter region, which is consistent with the increased recruitment of HDAC3 (Fig. 3o, p). These results collectively indicated that ZNF322A and HDAC3 transcriptionally downregulated *c-Myc* and suggested that c-Myc may exert a tumor suppressor function in the context of ZNF322A-mediated lung tumorigenesis or lung CSC-like reprogramming maintenance.

### c-Myc downregulation by ZNF322A overexpression helps maintain the metabolic phenotypes of CSCs

**Fig. 4:**
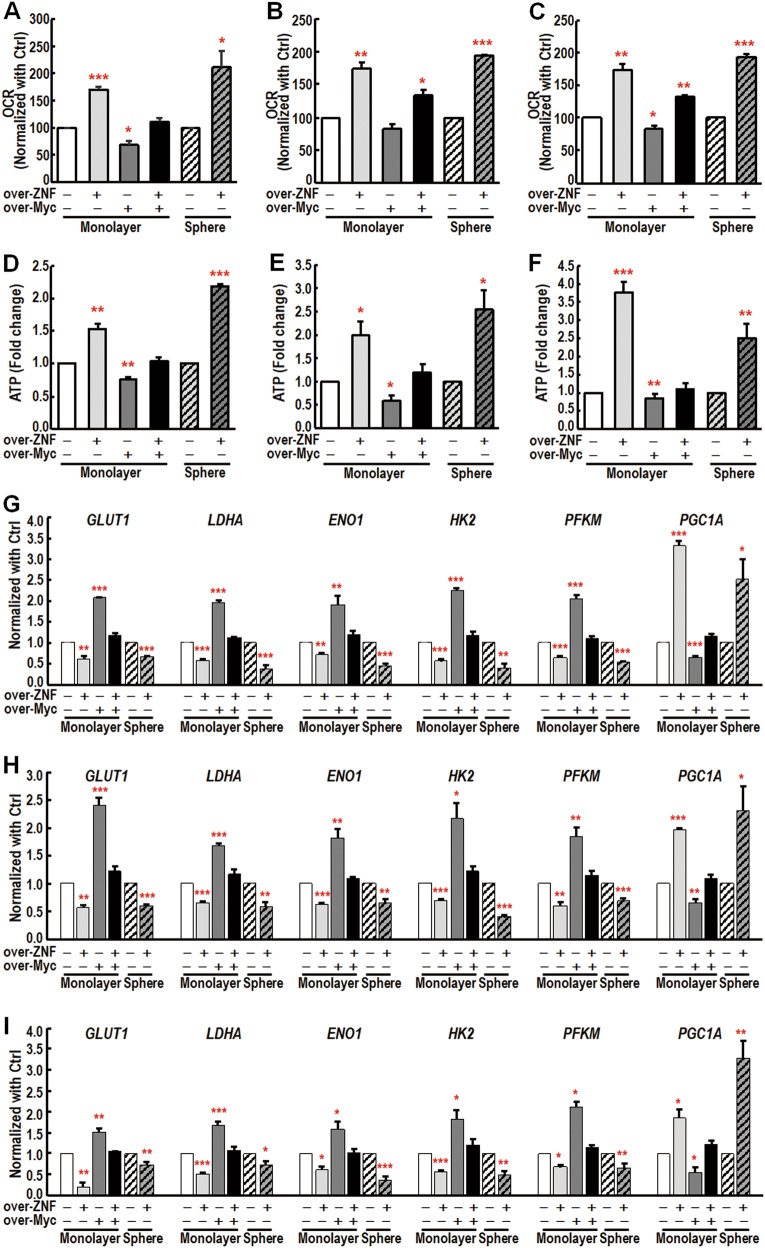
ZNF322A-mediated c-Myc suppression promotes metabolic reprogramming in lung cancer cells and lung cancer spheres. **a**–**c** Oxygen consumption rate (OCR) of over-ZNF cells, over-Myc cells or reconstituted cells (over-ZNF/over-Myc) or ZNF322A overexpressed spheres of H460 (**a**), H1299 (**b**), and A549 (**c**) was determined. **d**–**f** ATP content of over-ZNF cells, over-Myc cells or reconstituted cells (over-ZNF/over-Myc) or ZNF322A overexpressed spheres of H460 (**d**), H1299 (**e**), and A549 (**f**) was examined. **g**–**i** qRT-PCR analysis of metabolism-related genes in over-ZNF cells, over-Myc cells or over-ZNF/over-Myc cells or ZNF322A overexpressed spheres of H460 (**g**), H1299 (**h**), and A549 (**i**). The error bars represent SEM from three independent experiments (*P *<* 0.05; **P *<* 0.01; ***P *<* 0.001)

A recent study by Sancho and associates demonstrated that c-Myc expression is downregulated in most pancreatic CSCs compared with their cancer cell counterparts [17]. A distinct metabolic phenotype of mitochondrial oxidative phosphorylation in pancreatic CSCs was identified and c-Myc downregulation was the key determinant of dependency on oxidative phosphorylation to maintain full pancreatic CSC functionality [17]. Therefore, we examined mitochondrial activity by measuring the oxygen consumption rate (OCR) in ZNF322A overexpressed lung cancer cells and lung CSC-like cells with or without c-Myc reconstitution. The results showed that ZNF322A overexpression increased OCR in cells and spheres of H460, H1299, and A549, which was abolished upon c-Myc reconstitution (Fig. 4a–c). In concordance, ATP production was upregulated upon ZNF322A overexpression in cells and spheres, while c-Myc reconstitution suppressed ZNF322A-mediated ATP production (Fig. 4d–f). On the other hand, knockdown of c-Myc restored OCR and ATP production suppressed by ZNF322A knockdown (Supplementary Figure 5A–F).

Moreover, several c-Myc positively-regulated metabolic genes, including *GLUT1*, *LDHA*, *ENO1*, *HK2*, and *PFKM*, were downregulated upon ZNF322A overexpression in cells and spheres of H460, H1299 and A549. However, reconstitution of c-Myc restored expression of metabolic genes suppressed by ZNF322A in lung cancer cells (Fig. 4g–i). Notably, ZNF322A overexpression upregulated expression of the c-Myc negatively-regulated metabolic gene *PGC1A*, which is crucial for maintaining stemness properties of pancreatic CSCs [17]. In addition, ZNF322A-mediated *PGC1A* mRNA upregulation was abolished by c-Myc reconstitution (Figs. 4g–i). Importantly, c-Myc knockdown in ZNF322A-silenced lung cancer cells reversed the suppression of *LDHA* and *HK2* expression while reducing the upregulation of *PGC1A* (Supplementary Figure 5G–I). Together, these results indicated that ZNF322A-mediated c-Myc suppression plays an important role in sustaining CSC-like metabolism by maintaining mitochondrial oxidative phosphorylation and thereby maintaining lung CSC-like properties.

### ZNF322A-mediated c-Myc suppression promotes cancer cell motility

**Fig. 5:**
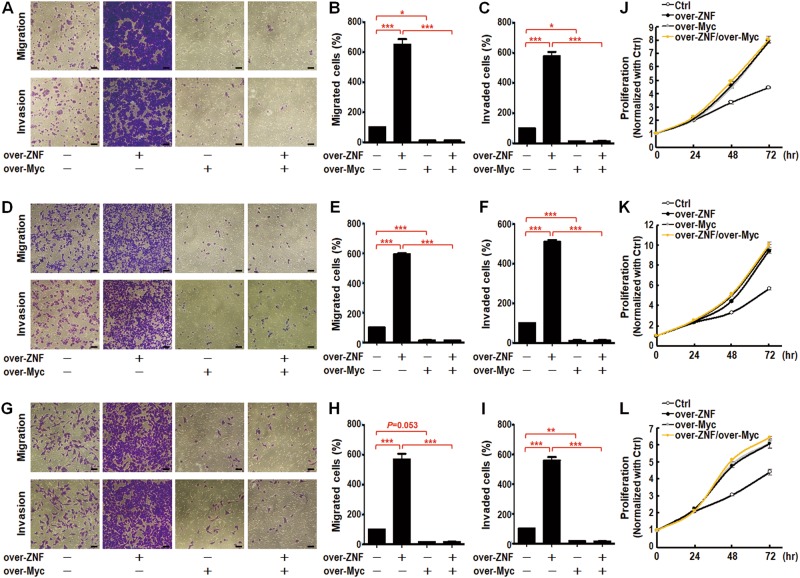
Reconstituted c-Myc expression reverses the oncogenic effects of ZNF322A on lung cancer cell motility but not on cell proliferation. **a**–**i** c-Myc overexpression suppressed cell migration and invasion abilities in ZNF322A overexpressing (over-ZNF/over-Myc) H460 (**a**-**c**), H1299 (**d**-**f**), and A549 (**g**–**i**) lung cancer cells. Scale bar, 200 nm. **j**–**l** c-Myc overexpression did not further promote cell proliferation abilities in ZNF322A overexpressing H460 (**j**), H1299 (**k**), and A549 (**l**) cells (over-ZNF/over-Myc *vs*. over-ZNF or over-Myc). Data are mean ± SEM (*P *<* 0.05; **P *<* 0.01; ***P *<* 0.001 as determined by one-way ANOVA)

Growing evidence has suggested the metastasis suppressor role of c-Myc in mouse skin and breast cancer [18–20]. c-myc causes a severe impairment in wound healing and in keratinocyte migration while promotes cell proliferation in *c-myc* transgenic mice [18, 19]. In addition, c-Myc overexpression suppresses cancer cell motility, invasion and metastasis by repressing transcription of α_v_β_3_ integrin [20]. These observations prompted us to investigate whether ZNF322A-mediated c-Myc suppression promoted lung cancer cell motility. We first performed transwell migration and invasion assay in lung cancer cells manipulated for c-Myc. The results showed that overexpression of c-Myc suppressed migration and invasion abilities in H460, H1299 and A549 cells at 16 h, whereas cell proliferation was enhanced at 48 and 72 h upon c-Myc overexpression. Downregulation of c-Myc exerted the opposite effects on the phenotype (Supplementary Figure 6). Next, we validated the motility suppressor role of c-Myc in the context of ZNF322A overexpression. Importantly, c-Myc overexpression abolished ZNF322A-promoted transwell migration and invasion abilities in multiple lung cancer cells (Fig. 5a–i) but not ZNF322A-promoted cell proliferation (Fig. 5j–l). Conversely, c-Myc knockdown restored migration suppression by ZNF322A knockdown (Supplementary Figure 7). Representative qRT-PCR results and immunoblots to confirm expression levels of ZNF322A and c-Myc in reconstitution studies are shown in Supplementary Figures 5 and 8. Our results confirmed the metastasis suppressor role of c-Myc in ZNF322A-mediated lung tumorigenesis.

### The negative correlation of ZNF322A and c-Myc expression was found in lung cancer patients and validated in TCGA public datasets

**Fig. 6:**
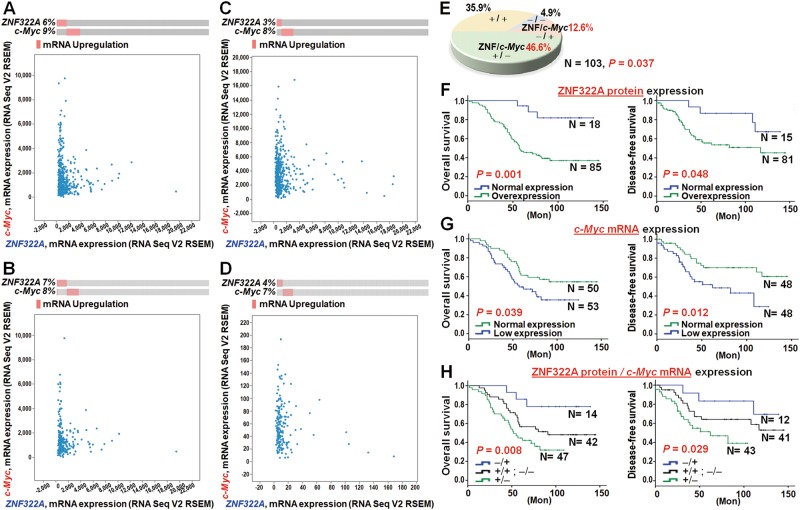
High ZNF322A protein expression correlates with low *c-Myc* mRNA expression and poor outcome in lung cancer patients. **a**–**d** Genetic alterations of *ZNF322A* and *c-Myc* in lung cancer patients from TCGA datasets. RNA-seq datasets for *ZNF322A* and *c-Myc* mRNA expression from two lung adenocarcinoma datasets, TCGA Provisional (**a**) and TCGA Nature 2014 [21] (**b**), and two lung squamous cell carcinoma datasets, TCGA Provisional (**c**) and TCGA Nature 2012 [22] (**d**), were extracted from TCGA through cBioPortal, and presented as OncoPrint (upper panel) and dot plots (lower panel) to emphasize mutual exclusivity. **e** Concordance analysis between ZNF322A protein and *c-Myc* mRNA expression (+, high expression; −, low expression) accordingly to the four molecular subtypes (−/−); (+/−); (+/+) and (−/+). ZNF322A protein expression is shown before the comma followed by *c-Myc* mRNA expression. The inversely correlated group included patients with (+/−) and (−/+) subtypes. *P* values determined using Pearson *χ*^2^-test. The percentage of cases is indicated on the pie chart. **f**–**h** Overall survival (left) and disease-free survival (right) analysis using Kaplan–Meier method indicated that patients with high ZNF322A protein expression (**f**), low *c-Myc* mRNA expression (**g**) and high ZNF/low Myc (+/−) expression (**h**) had poorer survival than other patients. *P* values determined using log-rank test

To further validate the inverse correlation between ZNF322A and c-Myc in lung cancer patients, four public lung cancer RNA-seq datasets using cBioPortal for The Cancer Genome Atlas (TCGA), including two lung adenocarcinoma datasets [21], and two lung squamous cell carcinoma datasets [22], were examined. Although the four public TCGA lung cancer datasets exhibited divergent levels of negatively correlated *ZNF322A* and *c-Myc* mRNA expression in the OncoPrint analyses, dot plot analyses for these four datasets showed significantly negative correlation (Fig. 6a–d).

In addition, we examined *c-Myc* mRNA expression and ZNF322A protein expression in 103 lung cancer patients to verify the clinically inverse correlation between ZNF322A and *c-Myc*. ZNF322A protein was found to be highly expressed in 82.5% of lung cancer patients while *c-Myc* mRNA was found to be expressed at a low level in 51.5% of lung cancer patients. Strikingly, an inverse correlation between ZNF322A protein and *c-Myc* mRNA in lung cancer patients was clearly evident (59.2%, *P* *=* 0.037, Fig. 6e).

### Lung cancer patients with ZNF322A^High^/c-Myc^Low^ expression profile correlate with poor prognosis

Since ZNF322A transcriptionally repressed *c-Myc* to promote CSC-like properties and cell motility, we hypothesized that expression of ZNF322A and *c-Myc* may be an effective prognotic biomarker in lung cancer. ZNF322A protein high expression in lung cancer patients was associated with poor overall survival (OS) and disease-free survival (Fig. 6f). *c-Myc* mRNA low expression was significantly associated with poor OS and DSF (Fig. 6g). Importantly, patients with ZNF322A protein overexpression and *c-Myc* mRNA low expression (ZNF322A^High^/*c-Myc*^Low^) showed worse OS and DSF compared with other patients (Fig. 6h).

To further determine whether ZNF322A^High^/*c-Myc*^Low^ expression profile served as an independent risk factor for poor outcome of lung cancer patients, we performed univariate and multivariate Cox regression analysis in 103 lung cancer patients. The univariate Cox regression analysis revealed that patients with ZNF322A^High^/*c-Myc*^Low^ expression profile and distant metastasis had poor outcome (*P* *=* 0.009, hazard ratio = 4.92, 95% confidence interval = 1.49–16.23 for ZNF322A^High^/*c-Myc*^Low^ expression). Of note, multivariate Cox regression analysis validated that ZNF322A^High^/*c-Myc*^Low^ expression exerted a significant hazard ratio of 6.49 (*P* *=* 0.011), even after adjusting with distant metastasis (Table 1). The clinical studies indicated that ZNF322A^High^/*c-Myc*^Low^ expression profile served as an independent risk factor for poor outcome of lung cancer patients.

**Table 1:** Cox regression analysis of risk factors for cancer-related death in lung cancer patients

Characteristics	Univariate analysis		Multivariate analysis	
	HR^a^ (95% CI^b^)	*P* value^c^	HR^a^ (95% CI^b^)	*P* value^c^
ZNF322A protein / *c-Myc* mRNA				
Low / High	1.00		1.00	
Other	2.93 (0.87–9.83)	0.082	3.69 (0.86–15.90)	0.080
High / Low	4.92 (1.49–16.23)	**0.009**	6.49 (1.53–27.46)	**0.011**
Age				
<65 year-old	1.00		–^e^	
≥65 year-old	0.68 (0.39–1.16)	0.154	–^e^	**–** ^**e**^
Gender				
Female	1.00		–^e^	
Male	1.41 (0.80-2.49)	0.239	–^e^	–^**e**^
Smoking habit				
Non-smoker	1.00		–^e^	
Smoker	1.32 (0.73-2.39)	0.361	–^e^	**–** ^**e**^
Tumor type^d^				
SCC	1.00		–^e^	
ADC	1.22 (0.68–2.21)	0.501	–^e^	**–** ^**e**^
Stage				
Stage I-II	1.00		–^e^	
Stage III-IV	1.40 (0.81–2.41)	0.229	–^e^	**–** ^**e**^
T stage				
T1-2	1.00		–^e^	
T3-4	1.41 (0.60–3.31)	0.428	–^e^	–^e^
N stage				
N0	1.00		–^e^	
≥N1	1.58 (0.91–2.74)	0.105	–^e^	–^e^
M stage				
M0	1.00		1.00	
≥M1	2.67 (1.24–5.73)	**0.012**	2.36 (1.10–5.08)	**0.028**

^a^*HR* hazard ratio

^b^*CI* confidence interval

^c^Bold values indicate statistical significance (*P* ***<*** 0.05)

^d^*AD* adenocarcinoma, *SCC* squamous cell carcinoma

^e^The variables without significant HR in the univariate analysis were not included in the multivariate analysis

## Discussion

**Fig. 7:**
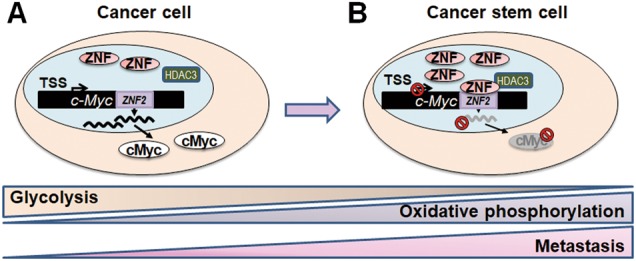
The schematic figure shows that ZNF322A promotes stem cell-like properties of lung cancer through transcriptional suppression of c-Myc expression. **a** In somatic lung cancer cells, ZNF322A expresses at a mediocre level so that c-Myc can be expressed. **b** ZNF322A expresses at high level in cancer stem-like cells, thus recruits HDAC3 to suppress c-Myc expression through targeting to ZNF322A binding motif 2 (ZNF2) on the *c-Myc* promoter. Moreover, ZNF322A-mediated c-Myc suppression shifts metabolism from glycolysis to mitochondrial oxidative phosphorylation and promotes cell motilities in lung cancer stem cells

Here, we characterized the transcriptional targets of ZNF322A using ChIP-seq and RNA-seq. We identified 128 positively-regulated genes and 118 negatively-regulated genes, and revealed novel roles of ZNF322A in the maintenance of CSC-like properties. Enforced ZNF322A expression alone induced CSC-like characteristics in multiple lung cancer cell lines. Notably, ZNF322A negatively regulated c-Myc expression at the transcriptional level to reprogram metabolism and promote cancer cell motility so as to maintain CSC-like properties and aggressiveness (Fig. 7). Moreover, an inverse correlation between ZNF322A and c-Myc was validated in our lung cancer patient cohort and public datasets from TCGA lung cancer cohorts. Collectively, our study provides the first evidence that ZNF322A transcriptionally reprogramed lung cancer cells into lung CSC-like cells, partly through negatively regulating c-Myc expression.

MEME motif analysis of our ChIP-seq datasets revealed DNA binding sequences of ZNF322A. Candidate genes in the RNA-seq provided targets to further validate the ZNF322A-mediated transcription through these canonical DNA binding elements. Promoter activity assay combined with site-directed mutagenesis demonstrated that ZNF322A top second-MEME motif (ZNF2) was crucial for ZNF322A-mediated downregulation of c-Myc transcription. Using this strategy, we found for the first time that ZNF322A bound directly to *c-Myc* promoter to transcriptionally suppress c-Myc expression. In our current ChIP-seq analysis, we mapped the ZNF322A targeting in genome and associated its bindings with the closest annotated gene. This ChIP-seq mapping strategy without taking into the consideration the enhancers or silencers involved may lead to low overlapping rate between ChIP-seq and RNA-seq datasets. Further ChIP-seq experiments by pull-down of enhancer markers, such as H3K27ac, H3K4me1 and MED1, and ATAC-seq will help establish a comprehensive transcription network of ZNF322A with higher overlapping rate with RNA-seq dataset.

Overexpressed ZNF322A bound to *c-Myc* and negatively regulated *c-Myc* transcription, which in turn increased mitochondrial oxidative phosphorylation. Although metabolic phenotypes of CSC are heterogeneous, lung CSCs and pancreatic CSCs appear to heavily depend on mitochondrial oxidative phosphorylation [17, 23, 24]. Sancho and associates reported that suppression of c-Myc increased the expression of its downstream gene PGC1A, a transcriptional co-activator of genes involved in oxidative phosphorylation, and thereby maintained cancer stemness in pancreatic CSCs [17]. We proposed that ZNF322A-mediated transcriptional downregulation of c-Myc may be required for shifting metabolism to oxidative phosphorylation to maintain ZNF322A-intitiated lung CSC-like cells. In agreement with our hypothesis, we identified a subgroup (46.6%) of lung cancer patients with ZNF322A^High^/*c- Myc*^Low^ expression profile manifesting poor outcome. This group of lung cancer patients could benefit from treatment with mitochondria-target agents such as metformin or menadione [25–28]. Alternatively, we proposed inhibitors of HDAC3 or knockdown of HDAC3 as chemotherapeutic strategies for lung cancer with ZNF322A^High^/*c-Myc*^Low^ expression. The sphere formation assay and *c-Myc* mRNA expression upon HDAC3 knockdown were assessed in H1299 cell lines. The preliminary results showed decrease in sphere size and number along with reactivation of *c-Myc* mRNA expression in HDAC3 knockdown compared to the control cells (Supplementary Figure 9). HDAC3-specific inhibitor has the potential to be tested for lung cancer treatment and combination chemotherapy.

c-Myc is a well-known oncogene which encodes a transcription factor that induces proliferation, transformation, metabolism and genomic instability in mammalian cells [29–31]. Although c-Myc-elicited proliferation is well-characterized [32–34], studies have also shown that c-Myc overexpression is dissociated with the propensity to invade and metastasize [35, 36]. For example, c-Myc overexpression is found to suppress the invasion and migration abilities of breast cancer cells by transcriptionally silencing the expression of α_v_ and β_3_ integrin subunits [20]. Consistently, our transwell migration and invasion assays confirmed that cells reconstituted with c-Myc suppressed ZNF322A-mediated lung cancer cell motility. Although previous studies have shown that transcription factors Oct4, Klf4, Sox2 and c-Myc together are capable of reprogramming embryonic fibroblasts back to pluripotent stem cells, c-Myc was later proved to be dispensable due to its role in enhancing proliferation rather than inducing pluripotency [37, 38]. These results collectively indicated that suppression of c-Myc by ZNF322A rendered CSC-like cells to be aggressively motile in the context of ZNF322A-mediated lung CSC-like properties maintenance.

In conclusion, we have uncovered a novel ZNF322A-center transcription program that induces lung CSC-like properties. To maintain CSC-like properties and full functionality, ZNF322A transcriptionally suppressed c-Myc expression to promote mitochondrial oxidative phosphorylation and cell motilities. Our mechanistic finding revealed that *c-Myc* promoter possesses a bone fide ZNF322A DNA binding element and could be repressed by ZNF322A corroborates with the clinical observation of the mutually exclusive expression between ZNF322A and c-Myc. Notably, ZNF322A^High^/*c-Myc*^Low^ expression profile in tumor specimen serves as an independent prognosis marker for poor outcome of lung cancer patients who may be considered for treatment strategy using mitochondria-targeting agents or HDAC3 inhibitors.

## Materials and methods

### Cell lines and culture

Human lung cancer cells H460, H1299 and A549 were purchased from American Type Culture Collection. A549 and H1299 cells were maintained in DMEM (Gibco, Grand Island, NY, USA), and H460 cells in RPMI1640 (Gibco). All media were supplemented with 10% fetal bovine serum (FBS) (Gibco) and 1% penicillin/streptomycin (Gibco). Cells were incubated at 37 °C in a humidified incubator containing 5% CO_2_.

### Chromatin-immunoprecipitation sequencing (ChIP-seq)

The chromatin-immunoprecipitation followed by deep sequencing (ChIP-seq) was performed in H460 cells expressing empty vector or *ZNF322A* expression vector. Cells were cross-linked and then lysed using Magna ChIP^TM^ protein G Kit (Millipore, Bedford, MA, USA) for nuclear extraction. Nuclear lysates were sonicated and then immunoprecipitated with anti-HA antibody (Abcam, Cambridge, MA, USA). Purified ChIP DNA was prepared for fragment libraries, which were subjected to high-throughput sequencing using a SOLiD^TM^ 5500xl sequencer (Applied Biosystems, Foster City, CA, USA). The raw reads were further analyzed using LifeScope^TM^ Genomic Analysis Software (version 2.5), and mapped to human genome (hg19) released from UCSC database [39]. The mapped profiles were analyzed using the ChIP-seq tool in CLC Genomics Workbench (version 4.9) with the human genome (hg19) as the default settings. Window size and false discovery rates were set to 200 bp and 5%, respectively. To determine the high confidence ZNF322A binding loci, the ChIP-region was identified by scanning the peaks with significantly higher read count in ZNF322A expressing cells compared to that in the control cells.

### De novo motif discovery and database matching of discovered motifs

For de novo motif discovery algorithm, MEME (Multiple Em for Motif Elicitation) was performed on ZNF322A ChIP-seq peaks for differentially expressed genes after ZNF322A manipulation in RNA sequencing (±50 bp from center of the ChIP-seq peak). To identify potential ZNF322A-interacting transcription factors, we compared the discovered motifs by MEME with two existing databases of known motifs, TRANSFAC (v11.3) and JASPAR (v3) using STAMP software. The TOMTOM and FIMO tool of the MEME Suite [40] (http://meme.ncbr.net/meme/cgi-bin/tomtom.cgi) were used to compare the similarity between predicted ZNF322A binding motifs from ChIP-seq and CASTing analyses, and to predict ZNF322A binding motifs from ChIP-seq on promoters of interest, respectively.

### Cyclic amplification and selection of targets (CASTing) in vitro oligo binding assay

To prepare a pool of random double-stranded oligonucleotides, random oligomers (5‵-GACTCGAGACTCCTAGGATGCGCA(N)_20_CGTCTATGTCAGTGAAGCTTCGAT-3‵) were incubated in PCR reaction buffer containing reverse primer (5‵-ATCGAAGCTTCACTGACATAGACG-3‵), deoxynucleoside triphosphates, and TITANIUM Tag DNA polymerase (ClonTech, Mountain Veiw, CA, USA), and amplified using the following cycling parameters: 5 min at 95 °C, 20 min at 65 °C and 20 min at 72 °C. Double-stranded random oligonucleotides were then incubated with GST-ZNF322A protein (Abnova, Walnut, CA, USA) bound to glutathione beads in a binding buffer containing 100 μg/ml poly(dI-dC). After 30 min of incubation on a rotating wheel, beads were washed with cold binding buffer without poly(dI-dC) and then boiled for 5 min. The eluted oligonucleotides were used for PCR amplification and for subsequent round of pulldown. PCR products were cloned into TOPO^®^ TA cloning vector (Invitrogen, Carlsbad, CA, USA), transformed into competent cells and sequenced after nine rounds of ZNF322A pulldown.

### Genome-wide RNA sequencing (RNA-seq)

Transcriptome libraries were prepared following the Applied BioSystems Library Builder^TM^ system according to the manufacturer’s instruction. The libraries were then sequenced using the Applied BioSystems 5500xl SOLiD Sequence. Mapping of sequencing reads and quantification of known RefSeq transcripts were performed using LifeScope^TM^ Genomic Analysis Software (version 2.5). Gene expression levels for each transcript were normalized using Reads Per Kilobase of exon model per Million mapped reads (RPKM) [41]. To determine the differentially expressed genes, we computed the *P* values by one-way ANOVA. We used *P* values < 0.05 and fold change > 1.25 as thresholds for determining differentially expressed genes between ZNF322A knockdown and knockdown control samples. Genes with statistically significant fold change in both ZNF322A positively-regulated or negatively-regulated groups were selected and biological processes of candidate genes were analyzed using MetaCore bioinformatic analysis software.

### Gene set enrichment analysis (GSEA)

GSEA was performed using ZNF322A knockdown gene set comprising differentially expressed genes with fold change greater than 1.25. Top 10% of differentially expressed genes on the microarrays of lung carcinoma tissues [16] or lung cancer cell lines (Wooster et al., not published) deposited in Oncomine (https://www.oncomine.org) or Zfp322a knockdown gene set in mouse embryonic stem cells [8] were ranked according to their differential expression levels across the two distinct phenotypes using a *t*-test metric. The *P* values were determined by a random permutation test.

### ChIP-qPCR and qRT-PCR assays

ChIP assays were performed using anti-HA, anti-ZNF322A, anti-HDAC3 or normal IgG in H460 cells expressing empty vector or *ZNF322A* expression vector. Antibodies are described in Supplementary Table 1. The DNA samples recovered from ChIP were analyzed by quantitative real time PCR using Fast SYBR Green Master Mix and StepOnePlus™ System (Applied Biosystems). For RNA expression assay, total RNA was extracted using Trizol reagent (Invitrogen, Carlsbad, CA, USA). Target gene expression levels were normalized to *GAPDH* expression levels. Primers used for qRT-PCR analysis are described in Supplementary Table 2.

### Tumor sphere formation assay

Cells transfected with empty vector or *ZNF322A* expression vector were expanded as spheres in 6-well ultra-low adhesion culture plate (Corning, New York, NY, USA) containing DMEM/F12 with N2 supplement (Invitrogen), 20 ng/ml epithelial growth factor and 20 ng/ml basic fibroblast growth factor (PeproTech Inc., Rocky Hill, NJ, USA). Tumor spheres consisting of >30 cells were photographed and counted.

### In vivo tumor initiation assay

Six-week-old female mice were obtained from National Cheng Kung University Laboratory Animal Center after obtaining appropriate institutional review board permission and mice were raised in pathogen-free conditions. Sphere cells (500 cells) from H460 transfected with empty vector or *ZNF322A* expression vector were suspended in a 1:1 dilution of Matrigel and implanted subcutaneously into BALB/c nude mice. Tumor initiation was checked every 3–4 days after injection. After 28 days, the mice were sacrificed and tumor tissues were fixed for histology studies.

### Dual luciferase promoter assay

Cells were plated in 12-well plates the day before transfection. The pGL3-Basic or pGL4-Renilla constructs were included as an internal control. After 16 h co-transfection with empty vector or various *c-Myc* promoter vectors, and pGL3-Basic or pGL4-Renilla, the dual luciferase reporter assay kit (Promega, Madison, WI, USA) was used to determine gene promoter activity according to the manufacturer’s protocol. The data are presented as the means of ratio of firefly luciferase to Renilla luciferase activity.

### XF extracellular flux analyzer experiments

Single cell suspensions of secondary spheres or transfected monolayer cells were seeded onto XF24 Cell Culture Microplates (Seahorse Bioscience, North Billerica, MA, USA) previously coated with Cell-Tak (BD Biosciences, San Jose, CA, USA) at a cell density of 1 × 10^5^ cells/well. The sensor cartridge was polarized overnight and calibrated. An hour before oxygen consumption rate (OCR) determination using XF Cell Mito Stress Kit (Seahorse Bioscience), the growth medium was replaced with the appropriate assay medium without sodium bicarbonate. The following compounds were injected sequentially: 0.5 μM oligomycin; 1 μM FCCP; 4 μM Rotenone. The basal OCR and OCR responses toward compounds injection were evaluated. All experiments were performed in triplicates.

### ATP production assay

Cellular ATP levels were measured using ATP Determination Kit (Molecular Probes, Eugene, OR, USA; A22066). ATP standard solutions were made by diluting 5 mM ATP in dH_2_O to concentrations of 1 nM to 1 μM. 10 μl of cell sample was then added to 100 μl standard reaction solution, and the luminescence was measured. The ATP standard curve was used to measure the ATP concentrations in cell samples, which was then normalized by the protein concentration.

### Transwell migration and invasion assay

The transwell insert with millipore membrane (pore size of 8 μm, Falcon, Lincoln Park, NJ, USA) was used. For transwell migration assay, 2 × 10^5^ cells were seeded onto the upper chamber. For invasion assay, the transwell insert membrane was pre-coated with Matrigel (2.5 mg/ml, Sigma-Aldrich) one day before cells were seeded. Complete medium containing 20% FBS was added to the lower chamber as chemoattractants and cells were incubated for 16 h. The cells attached on the reverse side of the membrane were then fixed and stained. Seven random views were photographed and quantified under an upright microscope (Nikon E400).

### RealTime-Glo viability assay

Cell viability was assayed using RealTime-Glo assay (Promega, Madison, WI, USA). Briefly, cells were transfected for 24 h and then reseeded at 2 × 10^3^ cells/well in 96-well plates. MT Cell Viability Substrate and NanoLuc Enzyme were diluted and added to each well. The luminescence was measured with a Turner BioSystems luminometer (Promega) at 24, 48, and 72 h.

### Study population

We recruited 103 Asian lung cancer patients from National Cheng Kung University Hospital, Taiwan after obtaining appropriate institutional review board permission and informed consent from the patients. Paraffin blocks of tumors were collected for immunohistochemical analysis of ZNF322A protein as previously reported [2]. qRT-PCR was used to analyze the expression of *c-Myc* in patient samples. *c-Myc* expression levels were normalized to *GAPDH* expression levels.

### Statistical analysis

Two-tailed Student’s *t*-test was used in all cell and animal studies unless otherwise indicated. Three independent experiments for cell studies and five mice per group for animal studies were analyzed unless indicated otherwise. Data represented as mean ± SEM. *P* *<* 0.05 was considered to be statistically significant.

### Data availability

The accession number for ChIP-seq dataset is GSE94656 and for RNA-seq dataset is GSE94537.

## Electronic supplementary material

CDD-18-0310_Supplementary information

CDD-18-0310_Table S4

CDD-18-0310_Table S5

CDD-18-0310_Table S6
