# Supplementary material for: Oncogenic zinc finger protein ZNF322A promotes stem cell-like properties in lung cancer through transcriptional suppression of c-Myc expression
Source: Cell Death Differ. 2018 Sep 26;26(7):1283–98. doi: 10.1038/s41418-018-0204-6 (PMC6748145; doi:10.1038/s41418-018-0204-6)
Supplement: Supplementary file 1 — CDD-18-0310_Supplementary information [file 41418_2018_204_MOESM1_ESM.pdf]

Supplementary Information

Oncogenic zinc finger protein ZNF322A promotes stem cell-like properties in lung cancer through transcriptional suppression of c-Myc expression

Jayu Jen, Chun-Yen Liu, Yu-Ting Chen, Li-Ting Wu, Yang-Chih Shieh, Wu-Wei Lai and Yi-Ching Wang

Inventory of supplementary information

Supplementary Data

Supplementary Figure 1 is related to Figure 1.
Supplementary Figure 2 is related to Figure 1.
Supplementary Figure 3 is related to Figure 2.
Supplementary Figure 4 is related to Figure 3.
Supplementary Figure 5 is related to Figure 4.
Supplementary Figure 6 is related to Figure 5.
Supplementary Figure 7 is related to Figure 5.
Supplementary Figure 8 is related to Figures 4 and 5.
Supplementary Figure 9 is related to Discussion.
Supplementary Table 1 is related to Materials and Methods.
Supplementary Table 2 is related to Materials and Methods.
Supplementary Table 3 is related to Materials and Methods.
Supplementary Table 4 is related to Figure 1.
Supplementary Table 5 is related to Figure 1.
Supplementary Table 6 is related to Figure 1.

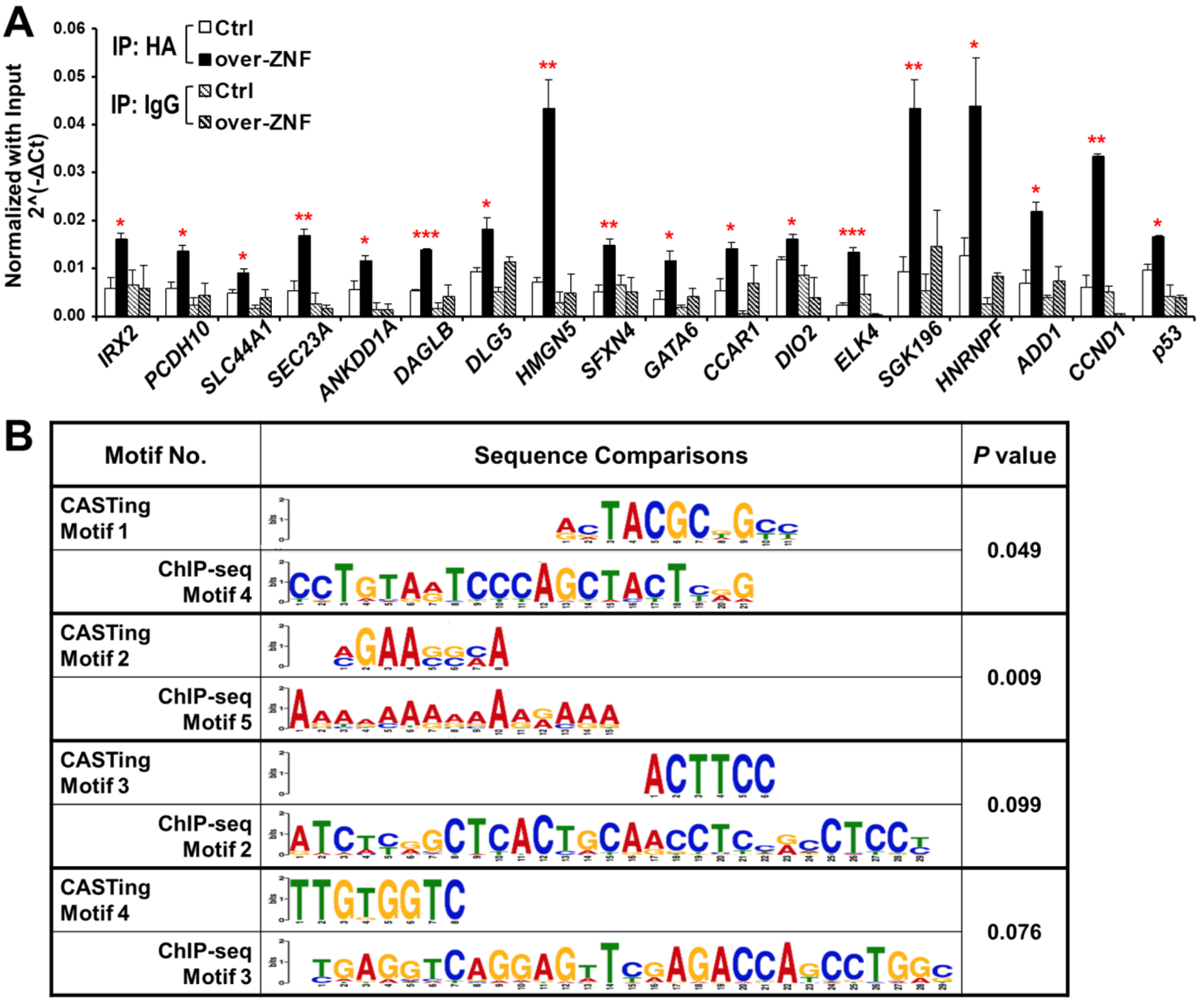

Supplementary Figure 1. Validation of ChIP-seq using ChIP-qPCR assay and comparisons of ZNF322A DNA binding elements with *in vitro* CASTing assay. A ChIP-qPCR analysis confirmed the HA-tagged ZNF322A occupancy at the binding sites of genes obtained from ChIP-seq dataset. ZNF322A-bound chromatin was immunoprecipitated using anti-HA antibody in H460 cells transfected with empty vector (Ctrl) or HA-tagged *ZNF322A* expression vector (over-ZNF). Normal IgG served as negative control. **B** MEME *de novo* elements prediction software was used to analyze the 101 oligonucleotides pulled down by 9 rounds of CASTing using GST-ZNF322A. The homology of predicted elements between CASTing assay and ChIP-seq were then analyzed using TOMTOM software. Similar sequences were aligned in the column "Sequence comparisons" with CASTing motif shown in the top and ChIP-seq in bottom. *P* value indicated the significant similarity of the motif between CASTing and ChIP-seq.

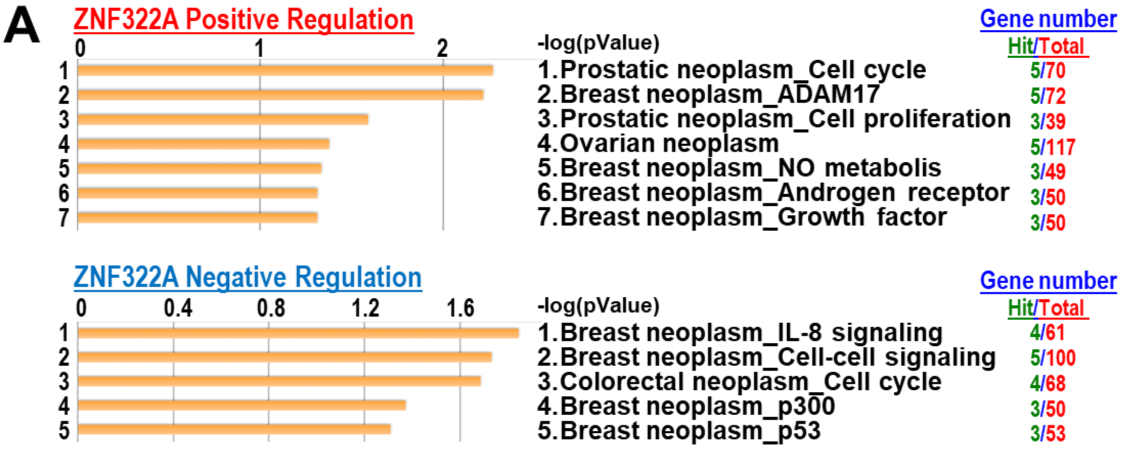

B

GO Function	Gene Count	*P*-value
• Cell morphogenesis	59	$1.1e^{-10}$
• Regulation of signal transduction	105	$2.2e^{-09}$
• Neuron projection morphogenesis	40	$4.9e^{-09}$
• Neuron development	53	$9.1e^{-09}$
• Neurogenesis	76	$5.6e^{-08}$
• Regulation of small GTPase mediated signal transduction	42	$6.6e^{-08}$
• Cell part morphogenesis	42	$1.0e^{-07}$
• Positive regulation of RNA metabolic process	60	$2.7e^{-06}$
• Positive regulation of transcription, DNA-dependent	59	$4.4e^{-06}$
• Cell migration	40	$5.3e^{-06}$
• Organ morphogenesis	65	$1.4e^{-05}$
• Blood vessel morphogenesis	32	$2.2e^{-05}$
• Blood vessel development	35	$3.1e^{-05}$
• Positive regulation of transcription	63	$4.9e^{-05}$
• Vasculature development	35	$5.1e^{-05}$
• Positive regulation of gene expression	64	$6.3e^{-05}$

Supplementary Figure 2. Disease biomarkers and gene ontology analyses of ZNF322A-driven transcriptome. A Disease biomarkers regulated by ZNF322A. ZNF322A transcriptional target genes, including 128 ZNF322A positively-regulated genes and 118 ZNF322A negatively-regulated genes, were analyzed by MetaCore resource. Top seven disease pathways mapped for genes from ZNF322A positively transcriptional target genes and top five from ZNF322A negatively transcriptional target genes are shown. **B** Gene ontology analysis of ZNF322A target genes by DAVID software.

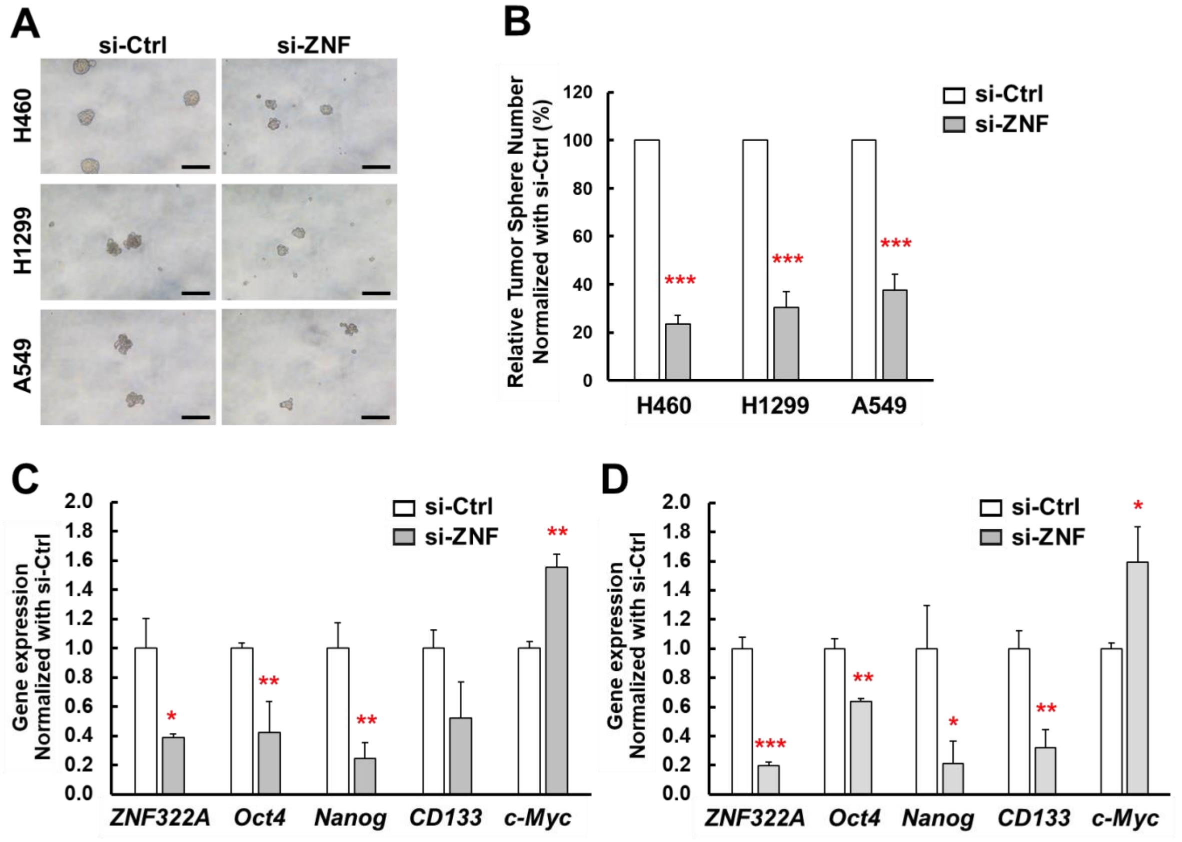

Supplementary Figure 3. Knockdown of endogenous ZNF322A suppresses self-renewal and stemness-related gene expression in lung cancer cells *in vitro*. A, B *In vitro* tumor sphere formation assay of H460, H1299 and A549 lung cancer cells expressing si-control (si-Ctrl) or si-*ZNF322A* (si-ZNF) oligos were photographed (**A**) and quantified (**B**). Scale bar, 200 nm. **C, D** qRT-PCR analysis of stemness-related genes and ZNF322A expression level in H460 (**C**) and H1299 (**D**) lung cancer cells expressing si-Ctrl or si-ZNF oligos. *GAPDH* was used as internal control. The error bars represent SEM from three independent experiments (**P* < 0.05; ***P* < 0.01; ****P* < 0.001).

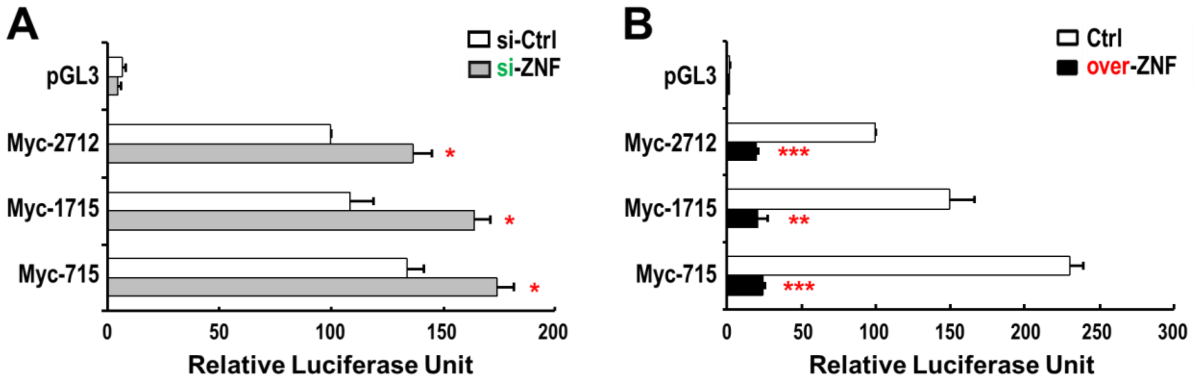

Supplementary Figure 4. ZNF322A negatively regulates *c-Myc* promoter activities. A, **B** Dual luciferase activity assays were performed using firefly luciferase reporter vectors containing -2157/+555, -1160/+555 or -160/+555 fragments of *c-Myc* promoters, which are indicated as Myc-2712, Myc-1715 and Myc-715, respectively. Data represent promoter activity in A549 cells expressing *ZNF322A* expression vector (over-ZNF) (**A**) or si-*ZNF322A* (si-ZNF) oligos (**B**). TSS: Transcription start site. Data are mean ± SEM (*$P < 0.05$; **$P < 0.01$; ***$P < 0.001$).

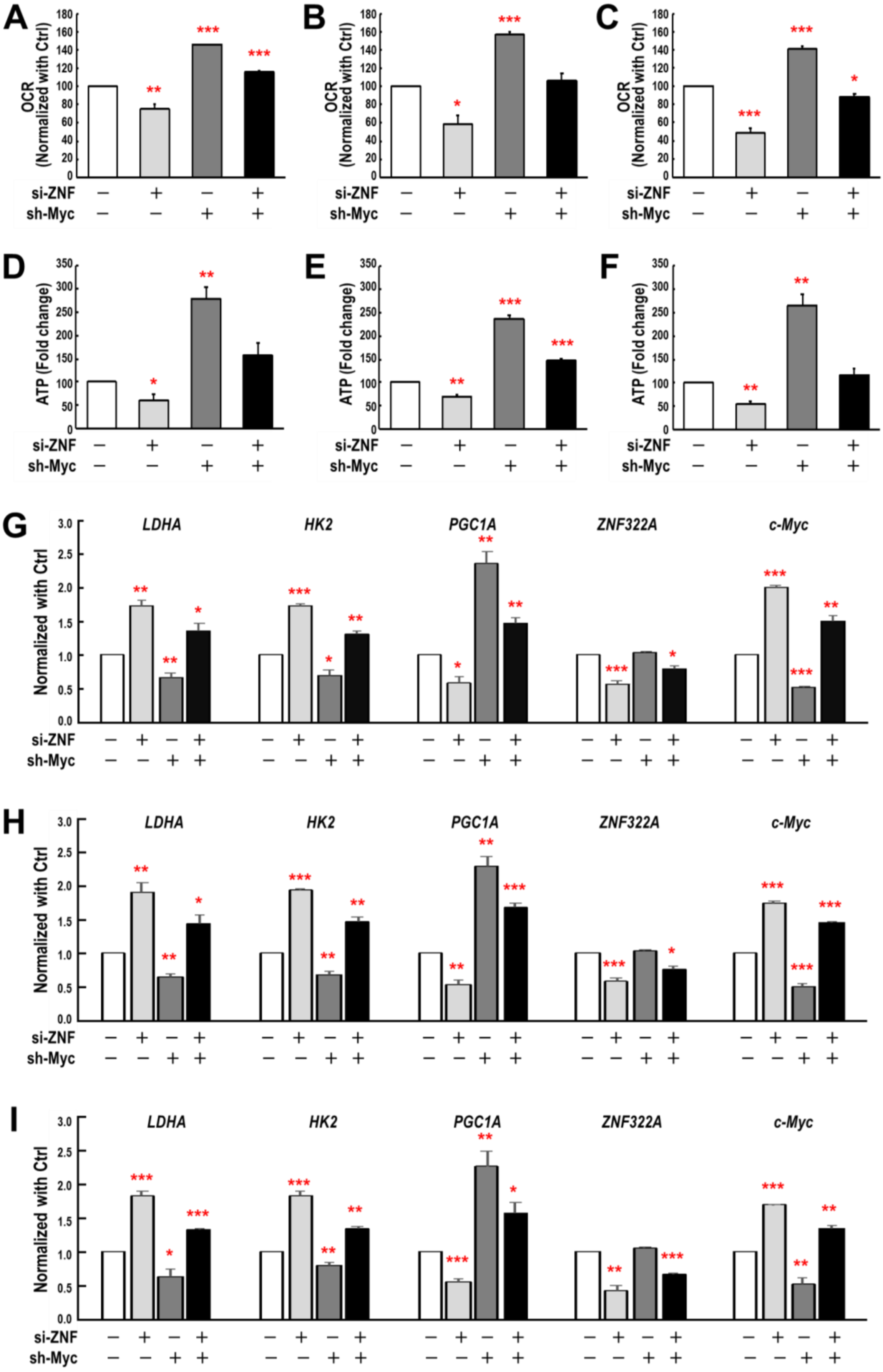

Supplementary Figure 5. Reconstitution experiments by c-Myc knockdown reversed

ZNF322A-mediated metabolic reprogramming in lung cancer cells. ZNF322A-mediated c-Myc suppression promotes metabolic reprogramming in lung cancer cells. **A**-**C** Oxygen consumption rate (OCR) was determined using XF Extracellular Flux Analyzer in single knockdown ZNF322A (si-ZNF), or c-Myc (sh-Myc) or in double knockdown reconstituted cells (si-ZNF/sh-Myc) of H460 (**A**), H1299 (**B**) and A549 (**C**) lung cancer cells. **D**-**F** ATP content of si-ZNF, sh-Myc, or si-ZNF/sh-Myc H460 (**D**), H1299 (**E**) and A549 (**F**) lung cancer cells was determined. **G**-**I** qRT-PCR analysis of metabolism-related genes in si-ZNF, sh-Myc, or si-ZNF/sh-Myc of H460 (**G**), H1299 (**H**) and A549 (**I**) lung cancer cells was examined. The error bars represent SEM from three independent experiments (**P* < 0.05; ***P* < 0.01; ****P* < 0.001).

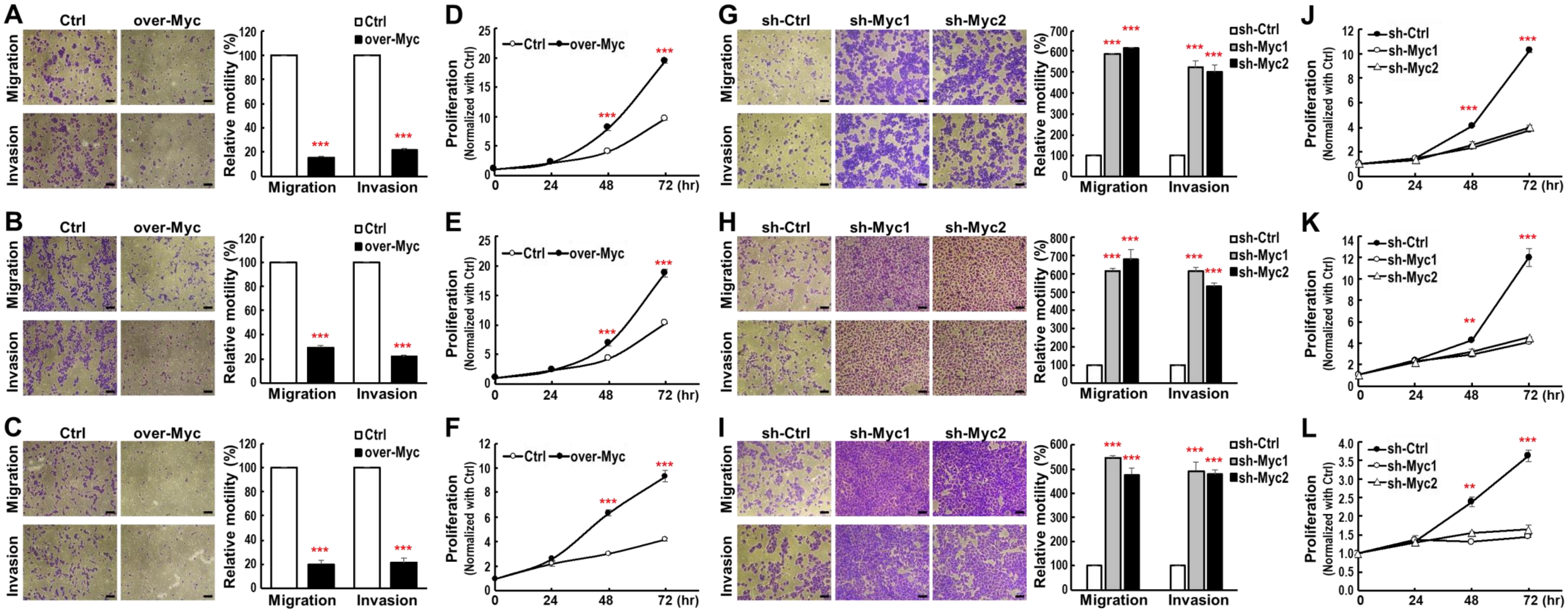

Supplementary Figure 6. c-Myc acts as a metastasis suppressor in lung cancer cells. A-C c-Myc overexpression (over-Myc) suppressed migration and invasion abilities in H460 (**A**), H1299 (**B**) and A549 (**C**) lung cancer cells. Represented figures (*left*) and quantified results (*right*) are shown. Scale bar, 200 nm. **D-F** c-Myc overexpression (over-Myc) promoted cell proliferation in H460 (**D**), H1299 (**E**) and A549 (**F**) lung cancer cells. **G-I** c-Myc silenced with two different shRNA clones (sh-Myc1 and sh-Myc2) increased migration and invasion abilities in H460 (**G**), H1299 (**H**) and A549 (**I**) lung cancer cells. Represented figures (*left*) and quantified results (*right*) are shown. Scale bar, 200 nm. **J-L** c-Myc silenced with two different shRNA clones (sh-Myc1 and sh-Myc2) suppressed cell proliferation in H460 (**J**), H1299 (**K**) and A549 (**L**) lung cancer cells. Data are mean ± SEM (**P < 0.01; ***P < 0.001).

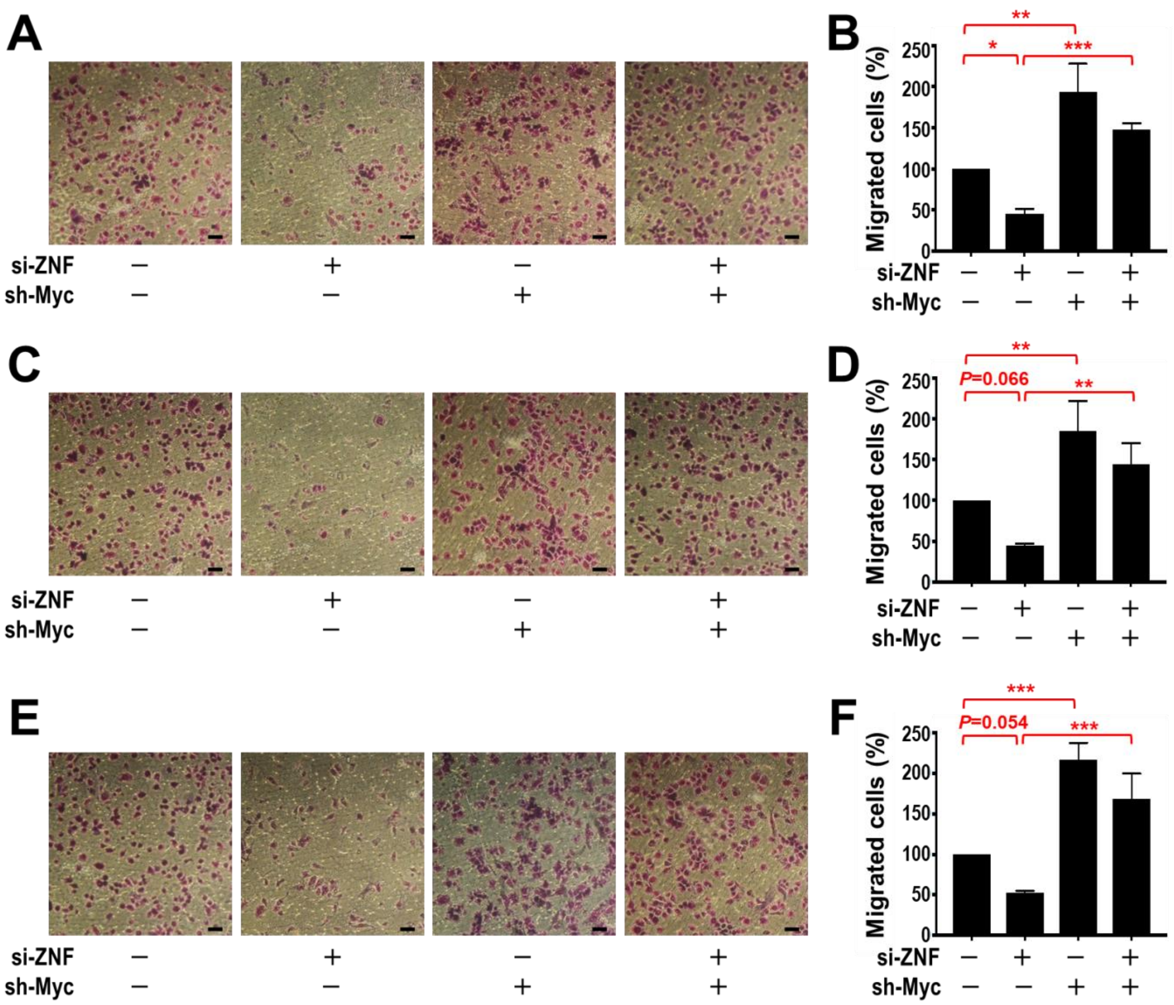

Supplementary Figure 7. Reconstitution experiments by c-Myc knockdown reversed ZNF322A-mediated migration in lung cancer cells. A-C c-Myc knockdown increased cell migration of ZNF322A depleted lung cancer cells (si-ZNF/sh-Myc) in H460 (**A**), H1299 (**B**) and A549 (**C**). Represented figures (*left*) and quantified results (*right*) are shown. Scale bar, 200 nm. Data are mean ± SEM (**P* < 0.05; ***P* < 0.01; ****P* < 0.001 as determined by One-Way ANOVA).

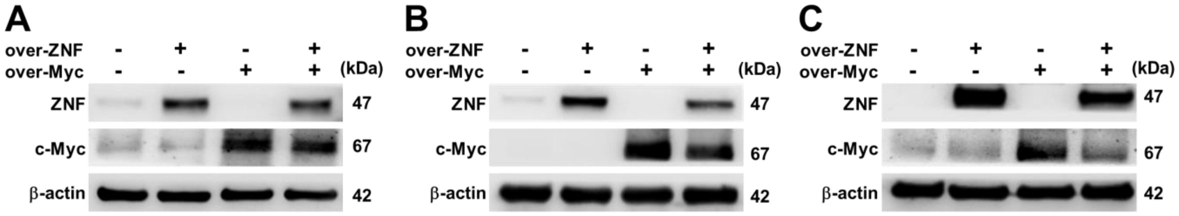

Supplementary Figure 8. Protein expression level of the manipulated ZNF322A and c-Myc in various lung cancer cells. A-C *ZNF322A* expression vector (over-ZNF) and *c-Myc* expression vector (over-Myc) were transfected into H460 (**A**), H1299 (**B**) and A549 (**C**) cells. Cell lysates were subjected to immunoblotting. β-actin serves as an internal control.

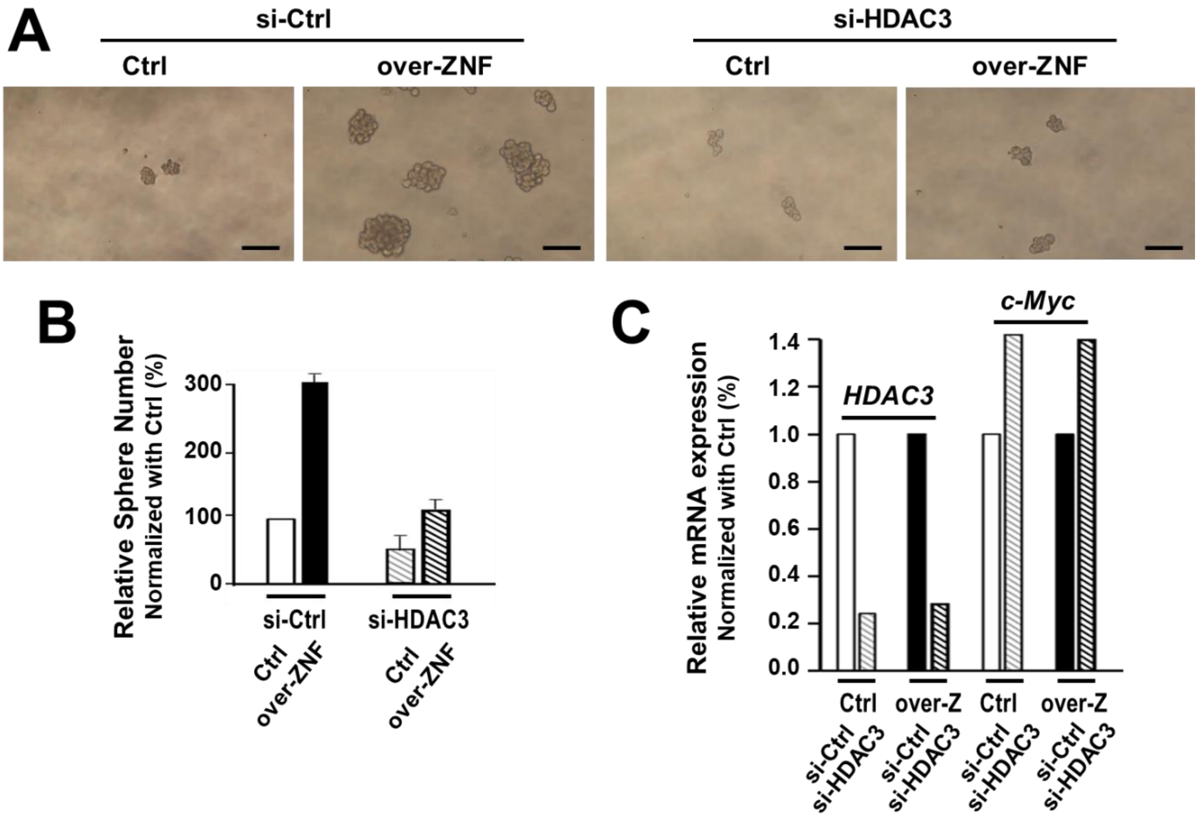

Supplementary Figure 9. Knockdown of HDAC3 reduces sphere formation and leads to re-expression of *c-Myc* mRNA. A, B H1299 cells overexpressing ZNF322A (over-ZNF) or empty vector (Ctrl) were transfected with control si-oligo (si-Ctrl) or mixed siRNA oligos against HDAC3 (recognize sequence 5-AGAGGCCUCAAACUUCUUGGCAUGG-3, 5-AACUCUGGUCAUCAAUGCCAUCCCG-3, 5-UUAAUUCUCCACAUCGCUUUCCUUG-3; # 1299001, Invitrogen, Carlsbad, CA, USA) then subjected for tumor sphere formation assay. Spheres were photographed (**A**) and quantified (**B**). Scale bar, 200 nm. **C** qRT-PCR analyses of *HDAC3* and *c-Myc* mRNA expression levels were measured in H1299 cells expressing ZNF322A (over-Z) and/or si-*HDAC3* (si-HDAC3) oligos.

Supplementary Table S1. Antibodies and their reaction conditions used in the current study

Target	KD	Raised In	Application	Dilution	Source	Catalog No.
ZNF322A	44	Rabbit	Immunoblotting<br>ChIP	1:1000<br>1:50	LifeSpan	LS-C30280
c-Myc	67	Mouse	Immunoblotting	1:1000	Santa Cruz	sc-42
β-actin	42	Mouse	Immunoblotting	1:5000	Novus Biologicals	NB 600-501
HDAC3	49	Rabbit	ChIP	1:50	Cell signaling	sc-11417
Ac-H3	16	Rabbit	ChIP	1:50	Millipore	06-599
HA-tag (ZNF322A)	–[a]	Rabbit	Immunoblotting<br>ChIP	1:1000<br>1:50	GeneTex	GTX29110

[a] –, Molecular weight is not applicable.

Supplementary Table S2. The primers used in the current study

Gene	Primer	Sequences (5'→ 3')	Application[a]	PCR size (bp)	Tm (°C)
GAPDH mRNA	Forward	GAG TCA ACG GAT TTG GTC GT	qRT-PCR	238	60
	Reverse	TTG ATT TTG GAG GGA TCT CG			
ZNF322A mRNA	Forward	GTG GTC TGC GTG TGA GAG TGG C	qRT-PCR	226	60
	Reverse	TTC TGA CGC ATG GGG AGG GCT			
ADD1 mRNA	Forward	TCC CAG GTT TTG TGT GGT GTA GT	qRT-PCR	150	60
	Reverse	ACG GTC TGT GAG TGT GGT GAA G			
CCND1 mRNA	Forward	AAC TAC CTG GAC CGC TTC CT	qRT-PCR	204	60
	Reverse	CCA CTT GAG CTT GTT CAC CA			
p53 mRNA	Forward	GTG GAA GGA AAT TTG CGT GT	qRT-PCR	184	60
	Reverse	CCA GTG TGA TGA TGG TGA GG			
DNAJC6 mRNA	Forward	CAC CAG AGC AGG TGA AGA AGG T	qRT-PCR	120	60
	Reverse	AGG CAT CAT TGA GCT CCA TGA			
GFRA3 mRNA	Forward	TTG CAG CTA AGA TGC GTT TTC A	qRT-PCR	103	60
	Reverse	GGC CTC ACA GCA GGG TTT T			
EDEM1 mRNA	Forward	CAA GAA TCC CTT CTA CCT CCA TGT A	qRT-PCR	131	60
	Reverse	GCT CTC CAT CCG GTC TTC TG			
KIF6 mRNA	Forward	GCA GAT TCG ATG TCT GTG ATG TG	qRT-PCR	133	60
	Reverse	ATG GAC GAC ACT GGC CTC TT			
IRX2 mRNA	Forward	GCC GGC GCC TCA AGA	qRT-PCR	170	60
	Reverse	GTC CAC GTG CAG GCT GAT C			
PCDH10 mRNA	Forward	GCC ATC GTC ACC GGT TAC AC	qRT-PCR	182	60
	Reverse	TCT CCA TGA CCA CTG TCC TTA GAG			
SLC44A1 mRNA	Forward	GAT AGT CTG CAG CAC AGG TTT AGC T	qRT-PCR	200	60
	Reverse	CAG GGC TCC CAT CAT TGT ATT T			

Gene	Primer	Sequences (5'→ 3')	Application[a]	PCR size (bp)	Tm (°C)
SEC23A mRNA	Forward	ACA GTG GCG GAA GTC AGG AT	qRT-PCR	181	60
	Reverse	TGA AGG GTT GAC TTT TGA AAG GA			
HBP1 mRNA	Forward	AAA TCC TGA CTG TTG GAA GAG GAA	qRT-PCR	161	60
	Reverse	GGT CAC ACG AAT TGA GGA CAA A			
CCR7 mRNA	Forward	TCC TTG TCA TTT TCC AGG TAT GC	qRT-PCR	101	60
	Reverse	GCA CAA AGA CTC GAA CAA AGT GTA G			
STXBP1 mRNA	Forward	GCA TTA CCA AGG CAC CGT AGA	qRT-PCR	130	60
	Reverse	GAC ATT GGC ATC CAG CAG AA			
ANKDD1A mRNA	Forward	TGG GAA GAG CTT GTC CTT TAA GC	qRT-PCR	177	60
	Reverse	CTG GTG CCT GTC CAC TGT TG			
ARHGAP23 mRNA	Forward	CCC CTG TGG GCG ACA A	qRT-PCR	124	60
	Reverse	GAC TTG GCT GAA CTA CAG GTG GTA			
DAGLB mRNA	Forward	TGC AGG AAT ATT CTC AGA GCT TCA	qRT-PCR	175	60
	Reverse	CCA AAC AGT TCG TAC CAC AAA CC			
DLG5 mRNA	Forward	ACA TTG CTC CGC ACG CTA TT	qRT-PCR	150	60
	Reverse	GCT CTT TGG AAT GCC TCT GAG T			
MYLK2 mRNA	Forward	GAC GGG ATC CTC TTC ATG CA	qRT-PCR	120	60
	Reverse	CCG TGC CAG GCC AAA GT			
PARD6G mRNA	Forward	CCC ACC ATA TCT CCA ACA GTG A	qRT-PCR	150	60
	Reverse	CGG CCT CCT CTC GTT TCT G			
MKNK1 mRNA	Forward	CAA CTC CTG TAC CCC CAT AAC C	qRT-PCR	120	60
	Reverse	CAG CGC TTG TCG TAG AAT GTG			
PAK4 mRNA	Forward	GCG CCC AGG TGA GCA A	qRT-PCR	151	60
	Reverse	TCT CCG TCC ACC ATC TCA ATC			
E2F2 mRNA	Forward	GAA GGC CAA GAA CAA CAT CCA	qRT-PCR	117	60
	Reverse	GCC TGC TCC GTG TTC ATC A			

Gene	Primer	Sequences (5'→ 3')	Application[a]	PCR size (bp)	Tm (°C)
KPNA6 mRNA	Forward<br>Reverse	CTG GGC CAT CAC CAA TGC<br>AGG ATG TTC TCC AGT CCA TTG AG	qRT-PCR	150	60
NMT1 mRNA	Forward<br>Reverse	TAT ACG CTG CCC TCC ACC AT<br>TCC ATG AGA TCC AGT GCA TTG	qRT-PCR	170	60
ZNF85 mRNA	Forward<br>Reverse	CTA GGT CTT GTT TTC CCT GCT TTG<br>CCA CAT CCC TAA ATG TCA ATG GT	qRT-PCR	150	60
HMGN5 mRNA	Forward<br>Reverse	GTC GTG CTG CAG TTA GTT CAT TG<br>ACT GGC ACA AGC ATA GCA GAC A	qRT-PCR	200	60
SFXN4 mRNA	Forward<br>Reverse	TTT CCA CAG ATT GGA CAG ATA CAG TAC T<br>CCA GAA GAC CTG TGG CAA AGT T	qRT-PCR	187	60
GATA6 mRNA	Forward<br>Reverse	GAA TTC AAA CCA GGA AAC GAA AAC<br>GGG AAG TAT TTT TGC TGC AAT CAT	qRT-PCR	135	60
NEFL mRNA	Forward<br>Reverse	CCA AGA CCC TGG AAA TCG AA<br>TCA CTC TTT GTG GTC CTC AAT TCA	qRT-PCR	151	60
CCAR1 mRNA	Forward<br>Reverse	AAG TCA TTG TGG TTA CCT TCT TGA AA<br>CTA GCA TAT CTT CCT GCA ATG ACT CA	qRT-PCR	200	60
NR1H4 mRNA	Forward<br>Reverse	TGC TCT GCT TAC AGC AAT TGT TAT C<br>CAG CGT GGT GAT GAT TGA ATG	qRT-PCR	200	60
ELK4 mRNA	Forward<br>Reverse	AGG CGC ATC GTG TTC GA<br>CTG CCT GCA AAA GCT TAA ACT G	qRT-PCR	177	60
ZNF238 mRNA	Forward<br>Reverse	TGC ATC TGT CTC TCT TAG TCT GCT TT<br>GTA GCA AAT GTC TAC TAT GGT CTG GAA	qRT-PCR	150	60
MXI1 mRNA	Forward<br>Reverse	GCC AAA GCA CAC ATC AAG AAA C<br>TCC AGT CGC CAC TTT AAA AAT CTC	qRT-PCR	101	60
SGK196 mRNA	Forward<br>Reverse	GCG AGG AGC TGA GAA CAG AAG<br>ATA GCC AAG CAG CGT GAC AA	qRT-PCR	200	60

Gene	Primer	Sequences (5'→ 3')	Application[a]	PCR size (bp)	Tm (°C)
HNRNPF mRNA	Forward	TCC AGA AGT GTC TCC CAC TGA A	qRT-PCR	107	60
	Reverse	CCC AGC ATC ATG GAC ACT TG			
GNB3 mRNA	Forward	GGG ACC TGC CGT CAG ACT T	qRT-PCR	175	60
	Reverse	TGA TGC CGC AGA TGA TGC T			
CTNNA3 mRNA	Forward	GCA AAG GAC CAC TAA AGC ATA CAA	qRT-PCR	152	60
	Reverse	CTG TTC CAG GTA GGC CAA CAA			
RIT1 mRNA	Forward	CAT GAT CCC ACC ATT GAA GAT G	qRT-PCR	175	60
	Reverse	AAC TTC GAC GAT CCG TGA TAG AG			
PFN2 mRNA	Forward	AGG AGG CCG CCA TTG TC	qRT-PCR	120	60
	Reverse	CTT CCC GGT CTT TTC CTA CAA TC			
CSNK2A2 mRNA	Forward	AGG CCA TGG AGC ACC CAT A	qRT-PCR	120	60
	Reverse	ACC CGT CGC TTT CCA GTC TT			
CAPN2 mRNA	Forward	TTG ATG ATG GAT TCA GGA GAC TGT	qRT-PCR	175	60
	Reverse	CCG TCC GAA TCT AGC ATG TCA			
Oct4 mRNA	Forward	CGA AAG AGA AAG CGA ACC AG	qRT-PCR	157	60
	Reverse	GCC GGT TAC AGA ACC ACA CT			
Nanog mRNA	Forward	CTG TGA TTT GTG GGC CTG AA	qRT-PCR	190	60
	Reverse	TCT TCC TTT TTT GCG ACA CTC TT			
Sox2 mRNA	Forward	ACA ACT CGG AGA TCA GCA	qRT-PCR	183	60
	Reverse	GCA GCG TGT ACT TAT CCT TC			
CD133 mRNA	Forward	ACA GTT TGC CCC CAG GAA AT	qRT-PCR	150	60
	Reverse	ATC CAT TCC CTG TGC GTT GA			
ABCB1 mRNA	Forward	GGT GCT GCT TTC CTG CTG AT	qRT-PCR	140	60
	Reverse	CCA ACA CTA AAA GCC CCA ATT AA			
ABCG2 mRNA	Forward	CCA TTG CAT CTT GGC TGT CA	qRT-PCR	180	60
	Reverse	CGA TGC CCT GCT TTA CCA AA			

Gene	Primer	Sequences (5'→ 3')	Application[a]	PCR size (bp)	Tm (°C)
c-Myc mRNA	Forward<br>Reverse	CTC TCC GTC CTC GGA TTC TCT<br>TTC CAC AGA AAC AAC ATC GAT TTC	qRT-PCR	157	60
HDAC3 mRNA	Forward<br>Reverse	AGC ACC CGC ATC GAG AAT C<br>GGA CAC TAG GTG CAT GGT TCA G	qRT-PCR	141	60
GLUT1 mRNA	Forward<br>Reverse	CGG GTT GTG CCA TAC TCA TG<br>CCA GTT GGA GAA GCC TGC AA	qRT-PCR	203	60
LDHA mRNA	Forward<br>Reverse	GAG AGT GCT TAT GAG GTG ATC AAA CT<br>CCA AAA TGC AAG GAA CAC TAA GG	qRT-PCR	181	60
ENO1 mRNA	Forward<br>Reverse	TGA ACG AGA AGT CCT GCA ACT G<br>TCT CCC CCG AAC GAT GAG	qRT-PCR	132	60
HK2 mRNA	Forward<br>Reverse	TCC GTA ACA TTC TCA TCG ATT TCA<br>CAG GTG CTC TCA AGC CCT AAG T	qRT-PCR	178	60
PFKM mRNA	Forward<br>Reverse	ACC AGA CAG ATT TTG AGC ATC GA<br>TAA TCT ATT CCC CTC ACT CCA GAG A	qRT-PCR	189	60
PGC1A mRNA	Forward<br>Reverse	TTG AAG AGC GCC GTG TGA T<br>CAG GTA TAA CGG TAG GTA ATG AAA CCA	qRT-PCR	160	60
ADD1	Forward<br>Reverse	GCT GCA GTG AGC CAT GGT T<br>TAT ATA CCC CAA AGA AAT GAA AAC AGC TA	ChIP-qPCR	180	60
CCND1	Forward<br>Reverse	GCC CAT TCT GCC GGC TTG GA<br>GGG GTG AGG TGG AGG TGG CT	ChIP-qPCR	132	60
p53	Forward<br>Reverse	GCA CCA GGT CGG CGA GAA TCC<br>TGC GAG GCT CCT GGC ACA AA	ChIP-qPCR	131	60
IRX2	Forward<br>Reverse	GCT TCT GGC AAA TTA AGA TCT CTC A<br>TCT GGT GGT GTG CAA CTG TAG TC	ChIP-qPCR	198	60
PCDH10	Forward<br>Reverse	GGA GGT TGC AGT AAG CCA AGA<br>GTG TTT TTG AAT TTT CCT TGA GAT GTC	ChIP-qPCR	186	60

Gene	Primer	Sequences (5'→ 3')	Application[a]	PCR size (bp)	Tm (°C)
SLC44A1	Forward<br>Reverse	TGA GAT GGA TTT TCA CTC GTG TTG<br>AGC ATG GAG AAA CCC TGT CTC TA	ChIP-qPCR	188	60
SEC23A	Forward<br>Reverse	GTG GAG ATC CAG TCT TTG TGT GAA<br>CAT GGG AGA TCA CAA ATA CAA GAA TAG	ChIP-qPCR	201	60
ANKDD1A	Forward<br>Reverse	AGT TCC AGG AGG ACG CTA AGC<br>TGG GCA AAA TGG TGA GAC CTA	ChIP-qPCR	200	60
DAGLB	Forward<br>Reverse	ATG TCC ACG TCA AGG GTT TTT T<br>CAC CCC TGG CTC ATT TTT GT	ChIP-qPCR	195	60
DLG5	Forward<br>Reverse	TCA TCC TGG AGG TGA GTT CTG A<br>GCT GTA CTC CTC CGT CTT TGG T	ChIP-qPCR	106	60
HMGN5	Forward<br>Reverse	CCC GCC TCC GCC TTT<br>GGT GCTA TTC GAG ATT CCC TAT TAA A	ChIP-qPCR	127	60
SFXN4	Forward<br>Reverse	CCT GGG AAG GTA GGT CAA AGC<br>CTT CCT GGC CTC ACG CAA T	ChIP-qPCR	206	60
GATA6	Forward<br>Reverse	CTT TTT TTG TTG AGA GGG AGT CTT G<br>ACA CGG TGA AAC TCG GTC TGT	ChIP-qPCR	185	60
CCAR1	Forward<br>Reverse	TGT GCT ATG ATT GCA CCT GTG A<br>GAC TGA CAG ACA TTC TCT GCT TGA C	ChIP-qPCR	177	60
DIO2	Forward<br>Reverse	GAC AAG GCA GGT GGA TCA TTG<br>GGC GCC ATC TCG CCT TA	ChIP-qPCR	196	60
ELK4	Forward<br>Reverse	GGA TCA TAG GAG TGA GCC AAT GT<br>TGG GAC GCT CGC TTG AG	ChIP-qPCR	127	60
SGK196	Forward<br>Reverse	GGA GTG CAA AGA AAA GAA GAA AAT AGA<br>ACA CAC AGA GAC TCG GGA GAA AC	ChIP-qPCR	125	60
HNRNPF	Forward<br>Reverse	CGA CGG AAA TGA GAC CTT GTC<br>AAC TCC CCA CAA GGA AGT CTT G	ChIP-qPCR	125	60

Gene	Primer	Sequences (5'→ 3')	Application[a]	PCR size (bp)	Tm (°C)
MYC	Forward	ACC CTT GCC GCA TCC A	ChIP-qPCR	186	60
	Reverse	CGT CTA AGC AGC TGC AAG GA			
ACTB	Forward	CAC CAC CAT GTA CCC TGG CAT	ChIP-qPCR	170	60
	Reverse	CAG TGA GGA CCC TGG ATG TGA C			
Myc-2712 promoter	Forward	GCG CAG ATC TAC TTT TTC CTC CAG TAA CTC CTC TT	Construction	--[b]	65
	Reverse	GCG CAA GCT TCT GGT TTT CCA CTA CCC G			
Myc-1715 promoter	Forward	GCG CAG ATC TGA GTT AAC GGT TTT TTT CAC AAG GG	Construction	--[b]	65
	Reverse	GCG CAA GCT TCT GGT TTT CCA CTA CCC G			
Myc-715 promoter	Forward	GCG CAG ATC TCG GCT GAG TCT CCT CCC C	Construction	--[b]	70
	Reverse	GCG CAA GCT TCT GGT TTT CCA CTA CCC G			
Myc-1-mut	Forward	GAA AAA AGA TCC TAG AGC GCT AAT CTC CGC	Site-direct mutagenesis	--[b]	71
	Reverse	GCG GAG ATT AGC GCT CTA GGA TCT TTT TTC			
Myc-2-mut	Forward	CTC TCT CGC TAA TAG AAG CCC ACC GGC CCT	Site-direct mutagenesis	--[b]	71
	Reverse	AGG GCC GGT GGG CTT CTA TTA GCG AGA GAG			
Myc+1-mut	Forward	CCA GGA CCC GCT TAG AGG AAA GGC TCT CCT	Site-direct mutagenesis	--[b]	71
	Reverse	AGG AGA GCC TTT CCT CTA AGC GGG TCC TGG			
Myc+2-mut	Forward	TCT CTG AAA GGC TAG AAT TGC AGC TGC TTA	Site-direct mutagenesis	--[b]	65
	Reverse	TAA GCA GCT GCA ATT CTA GCC TTT CAG AGA			

[a] qRT-PCR, quantitative reverse-transcriptase polymer chain reaction; ChIP-qPCR, quantitative chromatin-immunoprecipitation coupled with polymer chain reaction.

[b] --, Not applicable.

Supplementary Table S3. The plasmids and their characteristics used in the current study.

Plasmid	Target	Insert (bp)	Function	Source
pCMV-HA	None	–[a]	Vector control	Homemade
HA-ZNF322A	Wild type ZNF322A	1083	Overexpression	Homemade
pGL3-vector	None	–[a]	Vector control	Promega
pGL3-Myc-2712	*c-Myc* promoter from -2712 ~ -1	2712	Promoter activity assay	Homemade
pGL3-Myc-1715	*c-Myc* promoter from -1715 ~ -1	1715	Promoter activity assay	Homemade
pGL3-Myc-715	*c-Myc* promoter from -715 ~ -1	715	Promoter activity assay	Homemade
Myc-1-mut	Myc-715 promoter with ZNF322A binding element 2 mutation	715	Promoter activity assay	Homemade
Myc-2-mut	Myc-715 promoter with ZNF322A binding element 2 mutation	715	Promoter activity assay	Homemade
Myc+1-mut	Myc-715 promoter with ZNF322A binding element 2 mutation	715	Promoter activity assay	Homemade
Myc+2-mut	Myc-715 promoter with ZNF322A binding element 2 mutation	715	Promoter activity assay	Homemade

[a] –, Molecular weight is not applicable.

Supplementary Table S4. List for ZNF322A direct targeting genes is included in the excel file. The accession number for ChIP-seq dataset is GSE94656.

Supplementary Table S5. Gene list for 128 ZNF322A positively-regulated downstream targets is included in the excel file. The accession number for RNA-seq dataset is GSE94537.

Supplementary Table S6. Gene list for 118 ZNF322A negatively-regulated downstream targets is included in the excel file. The accession number for RNA-seq dataset is GSE94537.
